# Transition-metal-free synthesis of 3-sulfenylated chromones via KIO_3_-catalyzed radical C(sp^2^)–H sulfenylation

**DOI:** 10.3762/bjoc.13.199

**Published:** 2017-09-27

**Authors:** Yanhui Guo, Shanshan Zhong, Li Wei, Jie-Ping Wan

**Affiliations:** 1 College of Chemistry and Chemical Engineering, Jiangxi Normal University, Nanchang 330022, P.R. China

**Keywords:** C–H sulfenylation, chromones, domino reaction, free-radical, transition-metal-free

## Abstract

The reactions between *o*-hydroxylphenyl-functionalized enaminones and sulfonyl hydrazines providing 3-sulfenylated chromones via domino chromone ring construction and C(sp^2^)–H bond sulfenylation have been achieved under transition-metal-free conditions by using KIO_3_ as the only catalyst.

## Introduction

The C–S bond-forming reactions occupy a significant position in organic synthesis as they are the major route to install sulfur fragments to organic compounds. As prevalent substructures in many natural products and biologically relevant organic molecules, the C(sp^2^)–S bonds such as the C–S(sulfenyl) bond are known to play crucial roles in determining the properties and biological functions of sulfur-containing compounds [1–3]. Therefore, research works in developing efficient and flexible methodologies to generate C(sp^2^)–S bonds have attracted sustainable interest throughout the advances of modern organic synthesis. Presently, the most popular approaches in constructing a C(sp^2^)–S bond are the transition-metal-catalyzed Ullmann C–S coupling reaction [4–8], Chan–Lam cross-coupling reaction [9–12] as well as the transition-metal-catalyzed C–H bond activation [13–15].

**Scheme 1:**
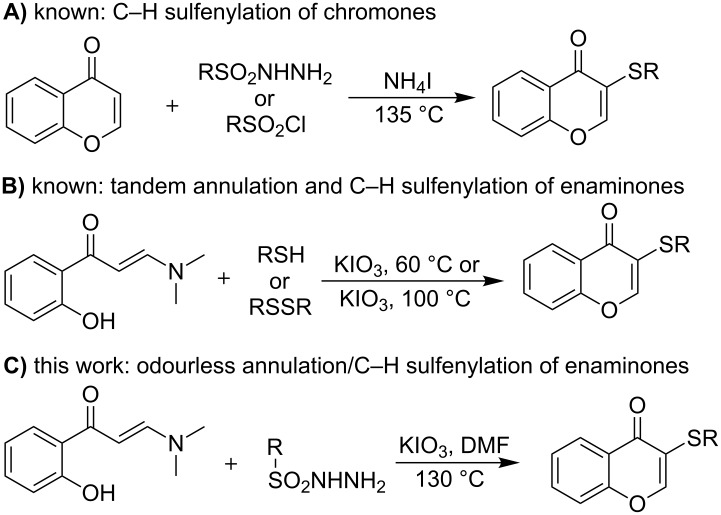
Methods on the synthesis of 3-sulfenylchromones.

Recently, as a new trend, the transition-metal-free oxidative coupling has emerged as a powerful complementary tactic in C(sp^2^)–S bond forming reactions [16–27]. As a beneficial and sustainable approach, such transition-metal-free cross coupling transformations have been successfully employed in the synthesis of many useful organic compounds elaborated with sulfenyl groups. For example, the 3-sulfenylated chromones, a class of useful organic molecules with important biological profiles [28–31] and extensive application in synthetic chemistry [32–37], have been readily synthesized with several different transition-metal-free C–H cross-coupling approaches. Zhou and co-workers reported the NH_4_I-promoted synthesis of 3-sulfenylated chromones via the direct chromone C–H sulfenylation by using different sulfur sources (A, Scheme 1) [38–39]. Recently, our continuous efforts in exploring enaminone C(sp^2^)–H bond sulfenylation [40–41] reactions have led us to establish the synthesis of 3-sulfenylated chromones via KIO_3_-catalyzed tandem reactions of *o*-hydroxylphenylenaminone and thiophenols via tandem C–H sulfenylation and intramolecular C–N bond oxygenation (B, Scheme 1) [42]. Subsequently, Braga et al. reported a similar catalytic 3-sulfenylchromone synthesis by employing disfulfides as the sulfenyl sources (B, Scheme 1) [43]. Although the efficiency and application scope of the known routes are fine, the cost and limited variation on chromone substrates and/or the utility of the odorous thiophenols remain as restrictions. In this regard, devising alternative synthetic methods featuring simultaneously the advantages of easily variable, low-cost substrates and operationally practical sulfur sources is highly demanding. Herein, we report a new synthetic protocol toward these compounds through the tandem reactions between *o*-hydroxyphenylenaminones and sulfonyl hydrazines. In this method, the construction of the target products is furnished via the key C–H sulfenylation without using any transition metal catalyst or oxidative additive.

## Results and Discussion

Initially, the reaction of enaminone **1a** and sulfonyl hydrazine **2a** was tentatively subjected to different iodine reagents or catalyst-free conditions. The results indicated that no product was observed in the reaction without catalyst (entry 1, Table 1), and different types of iodine reagents such as KI, I_2_ or KIO_3_ could all catalyze the domino reaction to provide product **3a**, wherein KIO_3_ displayed the highest catalytic activity (entries 2–4, Table 1). On the other hand, in the reactions performed in different media, including DMSO, ethyl lactate (EL), EtOH, MeCN, 1,4-dioxane and toluene, DMF was found as the most proper medium (entries 4–10, Table 1). Notably, increasing the reaction temperature to 130 °C promoted the production of **3a** with evidently higher yields (entries 11–13, Table 1). While varying the loading of KIO_3_ catalyst did not led to better results (entries 14 and 15, Table 1), prolonging the reaction time to 24 h was found to be capable of further improving the yield of **3a** (entries 16 and 17, Table 1).

**Table 1:** Optimization of the reaction conditions for the synthesis of sulfenylated chromones^a^.

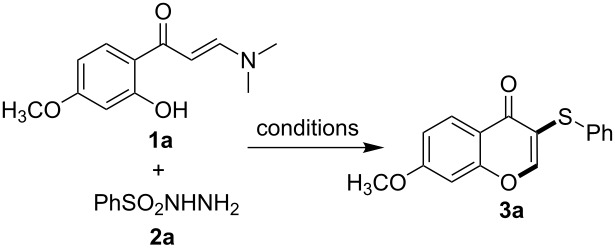				

Entry	Catalyst	Solvent	*T* (°C)	Yield (%)^b^

1	no	DMF	100	nr
2	KI	DMF	100	45
3	I_2_	DMF	100	56
4	KIO_3_	DMF	100	69
5	KIO_3_	DMSO	100	61
6	KIO_3_	EL	100	60
7	KIO_3_	EtOH	reflux	56
8	KIO_3_	MeCN	reflux	55
9	KIO_3_	1,4-dioxane	100	57
10	KIO_3_	toluene	100	62
11	KIO_3_	DMF	120	76
12	KIO_3_	DMF	130	79
13	KIO_3_	DMF	140	78
14^c^	KIO_3_	DMF	130	78
15^d^	KIO_3_	DMF	130	75
16^e^	KIO_3_	DMF	130	84
17^f^	KIO_3_	DMF	130	82

^a^General conditions: **1a** (0.3 mmol), **2a** (0.36 mmol), catalyst (0.15 mmol) in 2 mL solvent, stirred for 12 h (nr = no reaction). ^b^Yield of isolated product based on **1a**. ^c^The loading of KIO_3_ = 0.3 mmol. ^d^The loading of KIO_3_ = 0.09 mmol. ^e^The reaction time was 24 h. ^f^The reaction time was 30 h.

**Scheme 2:**
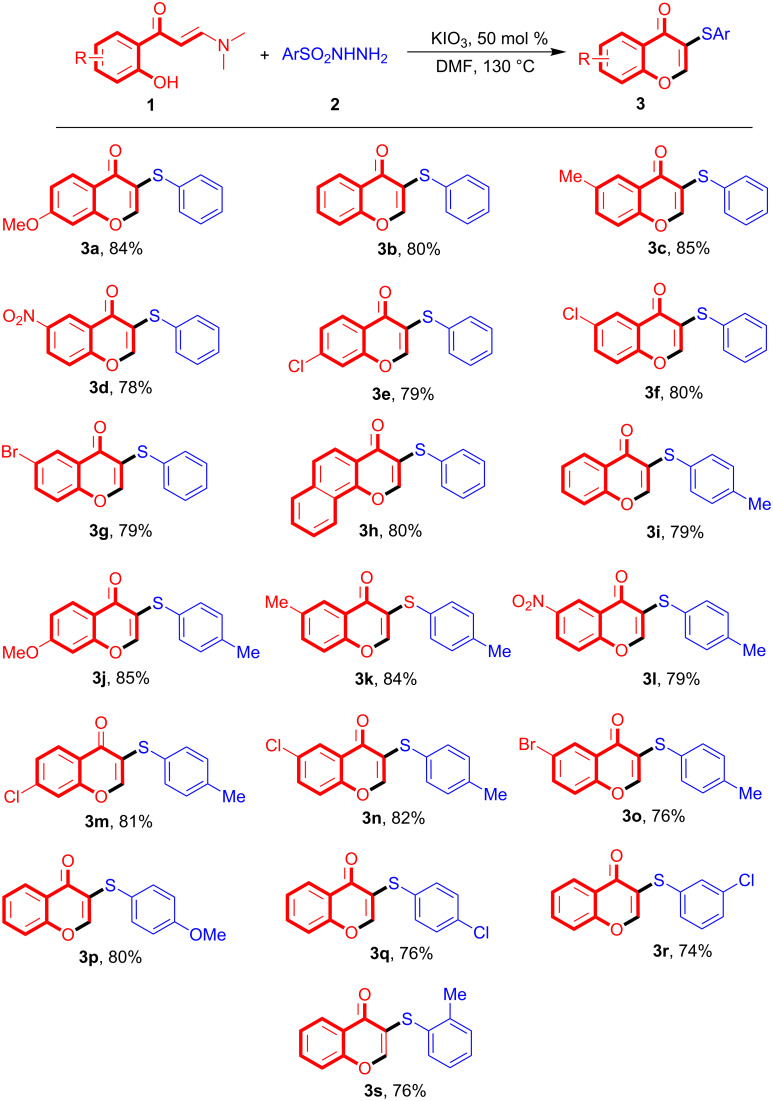
Scope of the 3-sulfenylated chromone synthesis. General conditions: **1** (0.3 mmol), **2** (0.36 mmol), KIO_3_ (0.15 mmol) in 2 mL DMF, stirred at 130 °C for 24 h; yield of isolated product based on **1** are reported.

To examine the scope of the reaction, enaminones **1** containing different functional groups as well as various sulfonyl hydrazines **2** were subjected to the optimized standard reaction conditions. As shown in Scheme 2, functional groups with different properties, including electron-withdrawing and electron-donating ones at the phenyl ring of the enaminones and the sulfonyl hydrazines exhibited tolerance to the reaction conditions. The 3-sulfenylated products were obtained in generally good to excellent yields. A strong electron-withdrawing group such as a nitro group at the phenyl ring of the enaminone was found to have a negative influence on the product yields (**3d** and **3l**, Scheme 2). On the other hand, the substituent on the aryl fragment of **2** also influenced the product yields. The main tendency was that a electron-withdrawing group on the aryl ring of the sulfonyl hydrazines and the group with *ortho*-steric hindrance gave the corresponding products with slightly lower yields than equivalent reactions using electron-donating-group-functionalized arylsulfonyl hydrazines (**3p**, **3q** and **3s**, Scheme 2).

**Scheme 3:**
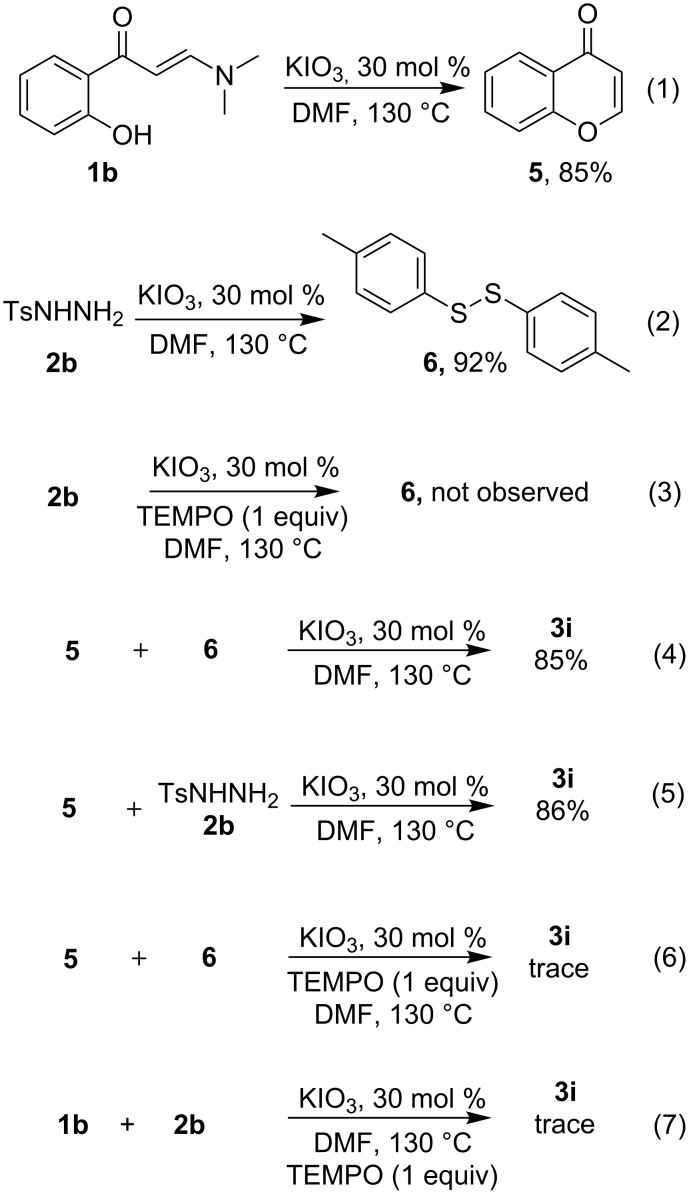
Control experiments.

On the basis of the results in hand, some control experiments were also designed to probe the possible reaction mechanism. As outlined in Scheme 3, directly using enaminone **1b** under standard conditions in the synthesis of products **3** was found to provide chromone **5** with high yield (reaction 1, Scheme 3). In addition, sulfonyl hydrazine **2b** gave disulfide **6** under the same conditions (reaction 2, Scheme 3), suggesting that chromone **5** and disulfide **6** might be key intermediates in the domino reactions. Moreover, the same reaction in the presence of TEMPO gave no formation of **6**, indicating that a free radical intermediate has also occurred in the generation of **6** (reaction 3, Scheme 3). The reactions of **5** with **6** and **2b** were both found to yield the sulfenylated chromone **3i** in excellent yield, respectively (reactions 4 and 5, Scheme 3). On the other hand, the reaction of **5** and **6** in the presence of TEMPO, however, provides only with trace amounts of **3i**, supporting that products **3** are yielded via a free radical route (reaction 6, Scheme 3). In addition, the control reaction of **1b** and **2b** afforded also only trace amounts of product **3i** in the presence of 1 equiv TEMPO (reaction 7, Scheme 3), further confirming that a radical intermediate was generated during the reaction process.

**Scheme 4:**
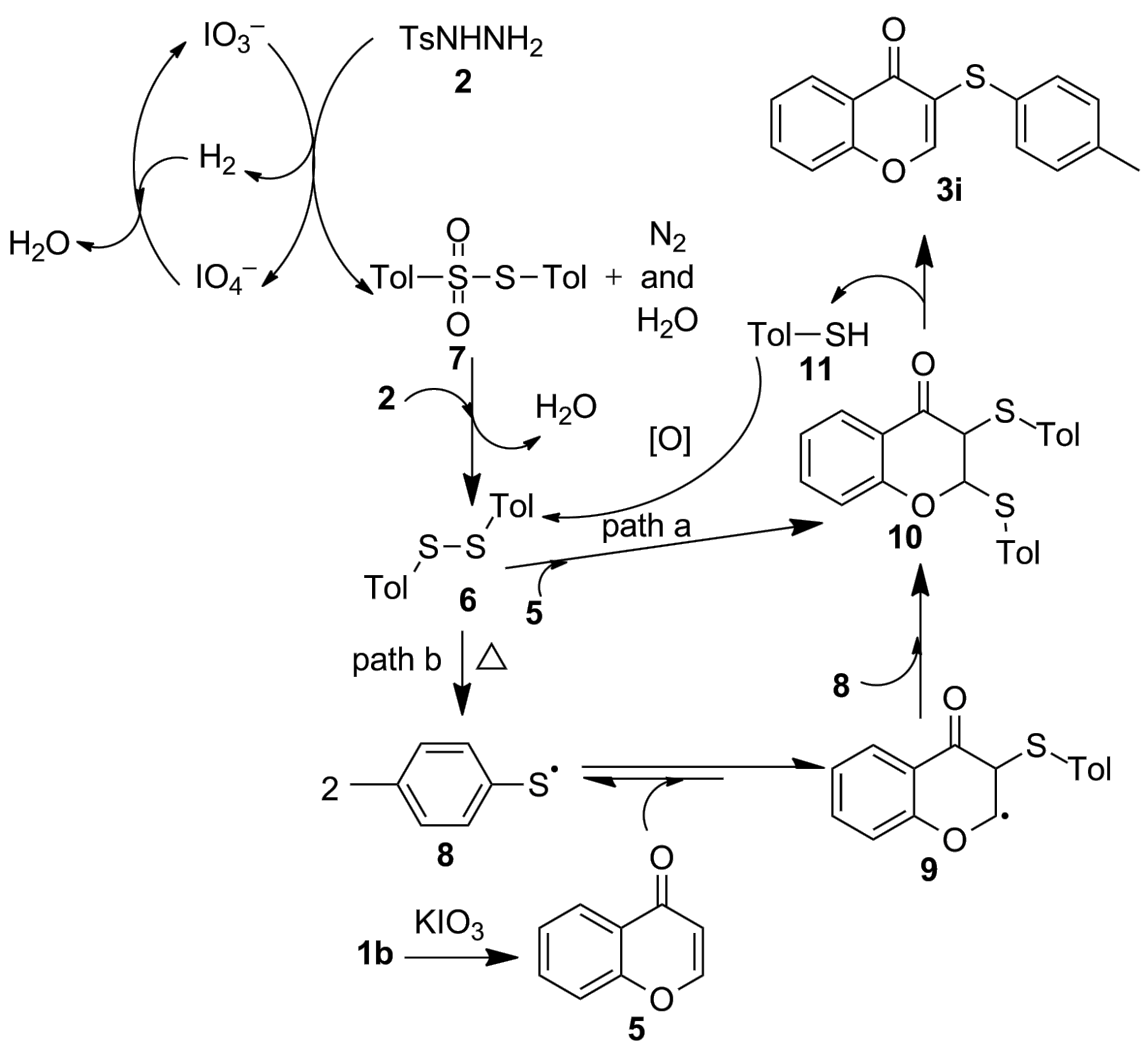
The proposed reaction mechanism.

According to the results obtained in the control experiments, the mechanism for this domino reaction is proposed (Scheme 4). As one of the key steps, the reductive coupling of two sulfonyl hydrazine molecules may first take place via the promotion of IO_3_^−^ to afford intermediate **7**, and the IO_4_^−^ generated therein returns to IO_3_^−^ in the presence of the simultaneously generated hydrogen in this step. According to a known route [44–45], **7** can be further reduced to yield disulfide **6**. The disulfide is capable of either coupling chromone **5** to generate intermediate **10** by radical chain propagation (path a), or transforming into sulfur radical **8** [46–47] which can be captured by the in situ generated chromone **5** to yield radical intermediate **9** via reversible addition to the double bond. The repeated coupling of **9** with radical **8** may also provide dithiolated intermediate **10** (path b). And the elimination of the α-hydrogen and β-sulfur gives target product **3i** and thiophenol **11**. The thiophenol could be easily reoxidized into disulfide **6** even under air atmosphere [48–49], which enables the recycled production of sulfenylated chromone. The present method involving the free radical pathway is different from our previous work on synthesizing identical products via an ionic-based mechanism.

## Conclusion

In conclusion, starting from *o*-hydroxyphenyl-functionalized enaminones and sulfonyl hydrazines, we have successfully developed a flexible and facile method for the synthesis of 3-sulfenylated chromones via a transition-metal-free reaction involving a C(sp^2^)–H bond sulfenylation. Besides the notable feature of transition-metal-free operation, the present method also displays advantages in using odourless sulfur sources, free of any strong oxidants and neutral reaction conditions.

## Supporting Information

File 1 General experimental information, experimental details on the synthesis of products **3**; full characterization data as well as ^1^H/^13^C NMR spectra of all products.
