# Supplementary material for: Transition-metal-free synthesis of 3-sulfenylated chromones via KIO3-catalyzed radical C(sp2)–H sulfenylation
Source: Beilstein J Org Chem. 2017 Sep 27;13:2017–22. doi: 10.3762/bjoc.13.199 (PMC5629394; doi:10.3762/bjoc.13.199)

Supporting Information

for

Transition-metal-free synthesis of 3-sulfenylated chromones via KIO_3-catalyzed radical $C(sp^2)$–H sulfenylation

Yanhui Guo[1], Shanshan Zhong[1], Li Wei[1] and Jie-Ping Wan*[1]

Address: [1]College of Chemistry and Chemical Engineering, Jiangxi Normal University, Nanchang 330022, P.R. China

Email: Jie-Ping Wan - wanjieping@jxnu.edu.cn

*Corresponding author

General experimental information, experimental details on the synthesis of products 3; full characterization data as well as $^1H/^{13}C$ NMR spectra of all products

Contents

General information……………………………………………………………….S2

General procedure for the synthesis of **3**……………….……………………………S2

Characterization data for products **3**…………………………………….……….S2–S8

References………………………………………………………………….....…….S8

^1H and ^{13}C NMR spectra of all products………………………...……..…….S9–S27

General information

All experiments were carried out under air atmosphere. All enaminones **1** were synthesized following a literature process,[1] and other chemicals and solvents used in the experiments were obtained from commercial sources and used directly without further treatment. ^{1}H and ^{13}C NMR spectra were recorded in a 400 MHz apparatus and the frequencies for ^{1}H NMR and ^{13}C NMR were 400 MHz and 100 MHz, respectively. The chemical shifts were reported in ppm with TMS as internal standard. Melting points were measured with a X-4A instrument and are uncorrected. The HRMS spectra were obtained in ESI mode with a TOF analyzer.

General procedure for the synthesis of 3-sulfenyl chromones 3. Into a 25 mL round-bottom flask were added enaminone **1** (0.3 mmol), sulfonyl hydrazide **2** (0.36 mmol), KIO_3 (0.15 mmol) and DMF (2 mL). Then the mixture was heated up to 130 °C, and stirred at the same temperature for 24 h (TLC). After cooling down to room temperature, 5 mL of water were added, and the resulting mixture was extracted with ethyl acetate (3 × 8 mL). The organic phases were combined and washed with small amount of water for three times. After drying with anhydrous Na_2SO_4, the solid was filtered and the solvent in the acquired solution was removed under reduced pressure. The resulting residue was subjected to flash silica gel column chromatography to provide pure products with the elution of mixed petroleum ether/ethyl acetate (v/v = 10:1).

Characterization data for products 3

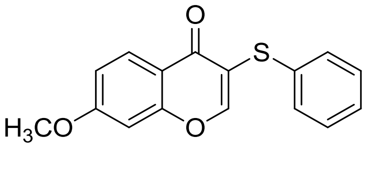

7-Methoxy-3-(phenylthio)-4*H*-chromen-4-one (3a).[2] Yield: 71 mg (84%); R_f = 0.3; white solid; m.p. 96-98 °C [lit. 98-100 °C]; ^{1}H NMR (400 MHz, $CDCl_3$): δ 8.15 (d, 1 H, *J* = 9.2 Hz), 8.09 (s, 1 H), 7.39-7.37 (m, 2 H), 7.30-7.26 (m, 2 H), 7.23-7.19 (m, 1 H), 7.99 (dd, 1 H, *J* = 8.8 Hz, 2.4 Hz), 6.84 (d, 1 H, *J* = 2.4 Hz), 3.91 (s, 3 H); ^{13}C NMR (100 MHz, $CDCl_3$): δ 174.4, 164.3, 158.2, 157.0, 134.3, 129.7, 129.2, 127.9,

127.0, 119.8, 117.6, 115.0, 100.3, 55.9; ESI-HRMS: Calcd for $C_{16}H_{13}O_3S$ $[M+H]^+$ 285.0580, found 285.0580.

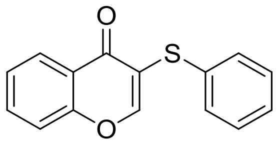

3-(Phenylthio)-4*H*-chromen-4-one (**3b**).[3] Yield: 61 mg (80%); R_f = 0.4; white solid; m.p. 98-100 ^oC [lit. 98-101 ^oC]; ^1H NMR (400 MHz, $CDCl_3$): δ 8.25 (dd, 1 H, *J* = 7.6 Hz, 1.2 Hz), 8.15 (s, 1 H), 7.71-7.67 (m, 1 H), 7.48-7.39 (m, 4 H), 7.30-7.28 (m, 2 H), 7.24-7.22 (m, 1 H); ^{13}C NMR (100 MHz, $CDCl_3$): δ 175.1, 157.4, 156.4, 134.0, 129.9, 129.2, 127.1, 126.5, 125.8, 123.7, 120.0, 118.2.

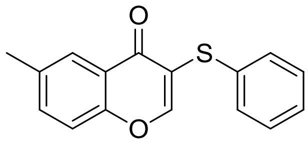

6-Methyl-3-(phenylthio)-4*H*-chromen-4-one (**3c**).[3] Yield: 68 mg (85%); R_f = 0.6; white solid; m.p. 105-107 ^oC [lit. 107-109 ^oC]; ^1H NMR (400 MHz, $CDCl_3$): δ 8.12 (s, 1 H), 8.00 (s, 1 H), 7.47 (d, 1 H, *J* = 8.4 Hz), 7.37-7.33 (m, 3 H), 7.25 (t, 2 H, *J* = 7.2 Hz), 7.18 (t, 1 H, *J* = 7.2 Hz), 2.42 (s, 3 H); ^{13}C NMR (100 MHz, $CDCl_3$): δ 175.1, 157.5, 154.6, 135.8, 135.3, 134.3, 129.6, 129.2, 127.0, 125.7, 123.4, 119.4, 117.9, 21.0.

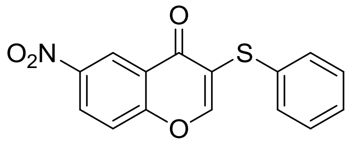

6-Nitro-3-(phenylthio)-4*H*-chromen-4-one (**3d**). Yield: 70 mg (78%); R_f = 0.3; yellow solid; m.p.147-149 ^oC; ^1H NMR (400 MHz, $CDCl_3$): δ 9.08 (d, 1 H, *J* = 2.8 Hz), 8.50 (dd, 1 H, *J* = 9.2 Hz, 2.8 Hz), 8.03 (s, 1 H), 7.63 (d, 1 H, *J* = 9.2 Hz), 7.45 (d, 2 H, *J* = 6.8 Hz), 7.36-7.27 (m, 3 H); ^{13}C NMR (100 MHz, $CDCl_3$): δ 173.6, 159.0, 155.8, 145.1, 132.1, 131.4, 129.5, 128.2, 128.1, 123.4, 123.1, 122.6, 120.1; ESI-HRMS: Calcd for $C_{15}H_{10}NO_4S$ $[M+H]^+$ 300.0325, found 300.0327.

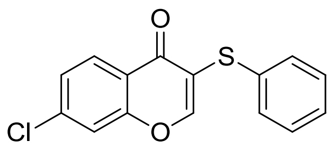

7-Chloro-3-(phenylthio)-4*H*-chromen-4-one (**3e**). Yield: 68 mg (79%); $R_f = 0.6$; white solid; m.p. 126-128 °C; ^{1}H NMR (400 MHz, $CDCl_3$): δ 8.16 (d, 1 H, *J* = 8.8 Hz), 8.05 (s, 1 H), 7.47 (d, 1 H, *J* = 1.2 Hz), 7.41-7.37 (m, 3 H), 7.31-7.28 (m, 2 H), 7.26-7.22 (m, 1 H); ^{13}C NMR (100 MHz, $CDCl_3$): δ 174.3, 156.7, 156.4, 140.1, 133.4, 130.4, 129.3, 127.8, 127.5, 126.6, 122.1, 121.0, 118.2; ESI-HRMS: Calcd for $C_{15}H_{10}ClO_2S$ $[M+H]^+$ 289.0085, found 289.0084.

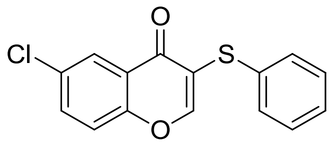

6-Chloro-3-(phenylthio)-4*H*-chromen-4-one (**3f**).[4] Yield: 69 mg (80%); $R_f = 0.6$; white solid; m.p. 133-135 °C; ^{1}H NMR (400 MHz, $CDCl_3$): δ 8.18 (d, 1 H, *J* = 2.4 Hz), 8.09 (s, 1H), 7.62 (dd, 1 H, *J* = 8.8 Hz, 2.4 Hz), 7.44-7.39 (m, 3 H), 7.31-7.22 (m, 3 H); ^{13}C NMR (100 MHz, $CDCl_3$): δ 174.0, 157.0, 154.7, 134.2, 133.4, 131.7, 130.3, 129.3, 127.5, 125.7, 124.5, 120.6, 119.9; ESI-HRMS: Calcd for $C_{15}H_{10}ClO_2S$ $[M+H]^+$ 289.0085, found 289.0083.

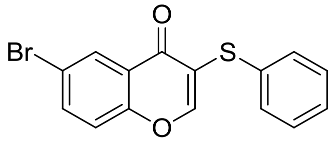

6-Bromo-3-(phenylthio)-4*H*-chromen-4-one (**3g**).[3] Yield: 79 mg (79%); $R_f = 0.5$; yellow solid; m.p. 118-120 °C [lit. 118-121 °C]; ^{1}H NMR (400 MHz, $CDCl_3$): δ 8.31 (d, 1 H, *J* = 2.0 Hz), 8.08 (s, 1 H), 7.73 (dd, 1 H, *J* = 8.8 Hz, 2.4 Hz), 7.39-7.34 (m, 3 H), 7.29-7.20 (m, 3 H); ^{13}C NMR (100 MHz, $CDCl_3$): δ 173.8, 157.0, 155.1, 137.0, 133.4, 130.3, 129.3, 128.9, 127.5, 124.8, 120.6, 120.2, 119.1.

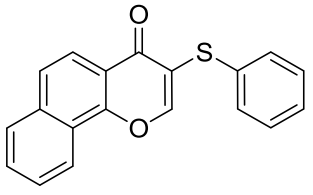

3-(Phenylthio)-4*H*-benzo[*h*]chromen-4-one (**3h**).[2] Yield: 73 mg (80%); $R_f = 0.2$;

white solid; m.p. 136-138 °C [lit. 117-119 °C]; ^{1}H NMR (400 MHz, $CDCl_3$): δ 8.40 (d, 1 H, *J* = 8.0 Hz), 8.18 (s, 1 H), 8.13 (d, 1 H, *J* = 8.8 Hz), 7.89 (d, 1 H, *J* = 8.0 Hz), 7.75-7.63 (m, 3 H), 7.47-7.45 (m, 2 H), 7.32-7.28 (m, 2 H), 7.26-7.24 (m, 1 H); ^{13}C NMR (100 MHz, $CDCl_3$): δ 174.8, 155.5, 153.8, 135.9, 133.5, 130.6, 129.6, 129.3, 128.2, 127.5, 127.4, 125.9, 123.8, 122.2, 122.1, 121.1, 119.8; ESI-HRMS: Calcd for $C_{19}H_{13}O_2S$ $[M+H]^+$ 305.0631, found 305.0631.

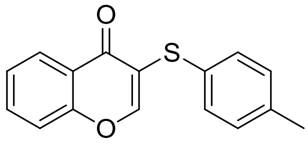

3-(*p*-Tolylthio)-4*H*-chromen-4-one (**3i**).[3] Yield: 63 mg (79%); white solid; R_f = 0.6; m.p. 106-108 °C [lit. 108-110 °C]; ^{1}H NMR (400 MHz, $CDCl_3$): δ 8.24 (d, 1 H, *J* = 7.6 Hz), 8.04 (s, 1 H), 7.68 (t, 1 H, *J* = 7.2 Hz), 7.46-7.40 (m, 2 H), 7.35 (d, 2 H, *J* = 7.6 Hz), 7.11 (d, 2 H, *J* = 7.6 Hz), 2.31 (s, 3 H); ^{13}C NMR (100 MHz, $CDCl_3$): δ 175.1, 156.3, 156.2, 137.6, 133.9, 131.0, 130.1, 129.8, 129.4, 125.6, 123.6, 121.1, 118.1, 21.1.

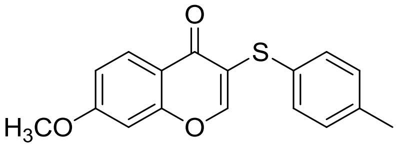

7-Methoxy-3-(*p*-tolylthio)-4*H*-chromen-4-one (**3j**).[3] Yield: 76 mg (85 %); R_f = 0.3; white solid; m.p. 124-126 °C [lit. 127-130 °C]; ^{1}H NMR (400 MHz, $CDCl_3$): δ 8.14 (d, 1 H, *J* = 8.8 Hz), 7.98 (s, 1H), 7.34 (d, 2 H, *J* = 8.0 Hz), 7.11 (d, 2 H, *J* = 8.0 Hz), 6.98 (dd, 1 H, *J* = 8.8 Hz, 2.0 Hz), 6.82 (d, 1 H, *J* = 2.0 Hz), 3.90 (s, 3 H), 2.31 (s, 3 H); ^{13}C NMR (100 MHz, $CDCl_3$): δ 174.4, 164.2, 158.1, 155.9, 137.5, 130.9, 130.1, 130.0, 127.7, 120.9, 117.5, 114.9, 100.3, 55.9, 21.1.

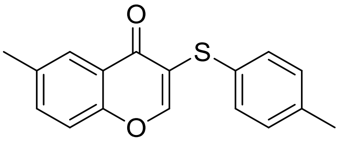

6-Methyl-3-(*p*-tolylthio)-4*H*-chromen-4-one (**3k**).[3] Yield: 71mg (84%); R_f = 0.6; white solid; m.p. 118-120 °C [lit. 117-120 °C]; ^{1}H NMR (400 MHz, $CDCl_3$): δ 8.00 (d, 2 H, *J* = 8.8 Hz), 7.45 (d, 2 H, *J* = 8.4 Hz), 7.32-7.27 (m, 3 H), 7.08 (d, 2 H, *J* = 7.6

Hz), 2.41 (s, 3 H), 2.28 (s, 3 H); ^{13}C NMR (100 MHz, $CDCl_3$): δ 175.1, 156.4, 154.6, 137.4, 135.7, 135.2, 130.8, 130.1, 130.0, 125.6, 123.2, 120.6, 117.9, 21.1, 21.0.

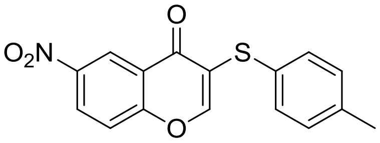

6-Nitro-3-(*p*-tolylthio)-4*H*-chromen-4-one (**3l**).[3] Yield: 74 mg (79%); R_f = 0.3; yellow solid; m.p. 155-158 °C [lit. 153-155 °C]; ^{1}H NMR (400 MHz, $CDCl_3$): δ 9.08 (d, 1 H, J = 2.8 Hz), 8.49 (dd, 1 H, J = 9.2 Hz, 2.8 Hz), 7.89 (s, 1 H), 7.61 (d, 1 H, J = 9.2 Hz), 7.40 (d, 2 H, J = 8.0 Hz), 7.16 (d, 2 H, J = 8.0 Hz), 2.34 (m, 3 H); ^{13}C NMR (100 MHz, $CDCl_3$): δ 173.6, 159.0, 154.6, 145.0, 138.8, 132.4, 130.4, 128.1, 127.8, 123.8, 123.2, 123.1, 120.0, 21.2.

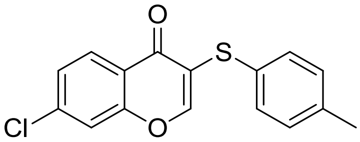

7-Chloro-3-(*p*-tolylthio)-4*H*-chromen-4-one (**3m**). Yield: 74 mg (81%); R_f = 0.7; white solid; m.p. 128-130 °C; ^{1}H NMR (400 MHz, $CDCl_3$): δ 8.14 (d, 1 H, J = 8.4 Hz), 7.92 (s, 1 H), 7.43 (s, 1 H), 7.35 (d, 3 H, J = 8.0 Hz), 7.11 (d, 2 H, J = 7.6 Hz), 2.31 (s, 3 H); ^{13}C NMR (100 MHz, $CDCl_3$): δ 174.2, 156.3, 155.5, 140.0, 138.0, 131.5, 130.2, 129.1, 127.7, 126.5, 122.1, 122.0, 118.2, 21.1; ESI-HRMS: Calcd for $C_{16}H_{12}ClO_2S$ $[M+H]^+$ 303.0241, found 303.0243.

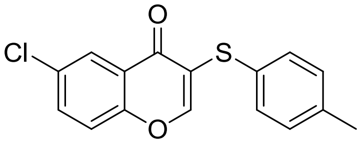

6-Chloro-3-(*p*-tolylthio)-4*H*-chromen-4-one (**3n**).[3] Yield: 75 mg (82%); R_f = 0.5; white solid; m.p. 175-178 °C [lit. 173-177 °C]; ^{1}H NMR (400 MHz, $CDCl_3$): δ 8.18 (d, 1 H, J = 2.4 Hz), 7.98 (s, 1 H), 7.60 (dd, 1 H, J = 8.8 Hz, 2.4 Hz), 7.41 (d, 1 H, J = 9.2 Hz), 7.35 (d, 2 H, J = 8.0 Hz),7.12 (d, 2 H, J = 8.0 Hz), 2.32 (s, 3 H); ^{13}C NMR (100 MHz, $CDCl_3$): δ 174.0, 155.8, 154.6, 138.0, 134.1, 131.6, 131.5, 130.2, 129.2, 125.7, 124.4, 121.8, 119.9, 21.1.

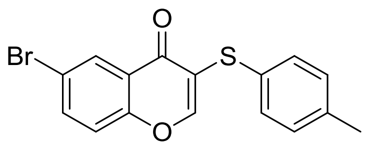

6-Bromo-3-(*p*-tolylthio)-4*H*-chromen-4-one (**3o**).[3] Yield: 79 mg (76%); R_f = 0.5; Yellow solid; m.p. 198-200 ^oC [lit. 199-202 ^oC]; ^1H NMR (400 MHz, $CDCl_3$): δ 8.35 (d, 1 H, *J* = 2.4 Hz), 7.98 (s, 1 H), 7.75 (dd, 1 H, *J* = 9.2 Hz, 2.8 Hz), 7.36-7.34 (m, 3 H), 7.12 (d, 2 H, *J* = 8.0 Hz), 2.32 (s, 3 H); ^{13}C NMR (100 MHz, $CDCl_3$): δ 173.8, 155.8, 155.1, 138.0, 136.9, 131.5, 130.2, 129.2, 128.9, 124.7, 121.9, 120.1, 119.0, 21.1.

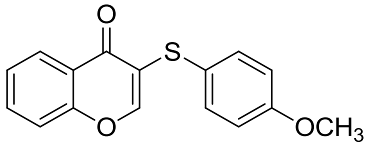

3-((4-Methoxyphenyl)thio)-4*H*-chromen-4-one (**3p**).[3] Yield: 68 mg (80%); R_f = 0.2; white solid; m.p. 110-113 ^oC [lit. 117-119 ^oC]; ^1H NMR (400 MHz, $CDCl_3$): δ 8.23 (d, 1 H, *J* = 9.6 Hz), 7.90 (s, 1 H), 7.68-7.64 (m, 1 H), 7.48 (d, 2 H, *J* = 8.8 Hz), 7.44-7.39 (m, 2 H), 6.86 (d, 2 H, *J* = 8.8 Hz), 3.79 (s, 3 H); ^{13}C NMR (100 MHz, $CDCl_3$): δ 175.1, 159.9, 156.3, 154.9, 134.3, 133.8, 126.3, 125.5, 123.4, 123.2, 122.6, 118.1, 115.0, 55.4.

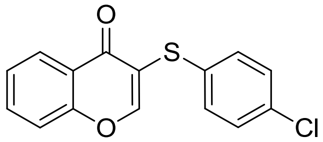

3-((4-Chlorophenyl)thio)-4*H*-chromen-4-one (**3q**).[3] Yield: 65 mg (76%); R_f = 0.3; white solid; m.p. 165-169 ^oC; ^1H NMR (400 MHz, $CDCl_3$): δ 8.25-8.23 (m, 2 H), 7.73-7.69 (m, 1 H), 7.49-7.43 (m, 2 H), 7.32 (d, 2 H, *J* = 8.4 Hz), 7.26-7.23 (m, 2 H); ^{13}C NMR (100 MHz, $CDCl_3$): δ 174.9, 157.8, 156.4, 134.1, 133.2, 132.8, 131.0, 129.3, 126.5, 125.9, 123.8, 119.3, 118.2.

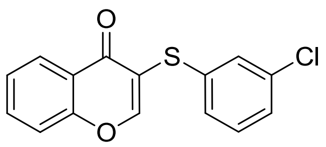

3-((3-Chlorophenyl)thio)-4*H*-chromen-4-one (**3r**). Yield: 64 mg (74%); R_f = 0.4; white solid; m.p. 128-130 ^oC; ^1H NMR (400 MHz, $CDCl_3$): δ 8.27 (s, 1 H), 7.24 (d, 1

H, J = 8.0 Hz), 7.71 (t, 1 H, J = 7.2 Hz), 7.50-7.43 (m, 2 H), 7.30 (s, 1 H), 7.26-7.23 (m, 1 H), 7.21-7.14 (m, 2 H); ^{13}C NMR (100 MHz, $CDCl_3$): δ 174.8, 158.5, 156.4, 136.7, 134.9, 134.2, 130.1, 128.6, 127.1, 127.0, 126.5, 126.0, 123.8, 118.5, 118.2; ESI-HRMS: Calcd for $C_{15}H_{10}ClO_2S$ $[M+H]^+$ 289.0085, found 289.0085.

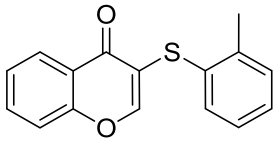

3-(*o*-Tolylthio)-4*H*-chromen-4-one (**3s**).[3] Yield: 61 mg (76%); R_f = 0.4; white solid; m.p. 156-159 °C [lit. 154-155 °C]; ^{1}H NMR (400 MHz, $CDCl_3$): δ 8.25 (dd, 1 H, J = 8.0 Hz, 1.2 Hz), 7.86 (s, 1 H), 7.68 (t, 1 H, J = 6.8 Hz), 7.46-7.41 (m, 2 H), 7.27-7.22 (m, 2 H), 7.19-7.15 (m, 1 H), 7.13-7.09 (m, 1 H), 2.49 (s, 3 H) ; ^{13}C NMR (100 MHz, $CDCl_3$): δ 175.0, 156.4, 155.5, 138.9, 133.9, 132.2, 131.0, 130.7, 127.6, 126.8, 126.4, 125.6, 123.4, 120.5, 118.1, 20.4.

References

1) El-Taweel, F. M. A. A.; Elnagdi, M. H. J. *Heterocyclic Chem*. **2001**, *38*, 981.

2) Rafique, J.; Saba, S.; Schneider, A. R.; Franco, M. S.; Silva, S. M.; Braga, A. L. *ACS Omega* **2017**, *2*, 2280.

3) Zhong, S.; Liu, Y.; Cao, X.; Wan, J.-P. *ChemCatChem*. **2017**, *9*, 465.

4) Zhao, W.; Xie, P.; Bian, Z.; Zhou, A.; Ge, H.; Zhang, M.; Ding, Y.; Zheng, L. *J. Org. Chem.* **2015,** *80*, 9167.

^{1}H and ^{13}C NMR spectra of all products

^{1}H and ^{13}C NMR spectra of **3a**

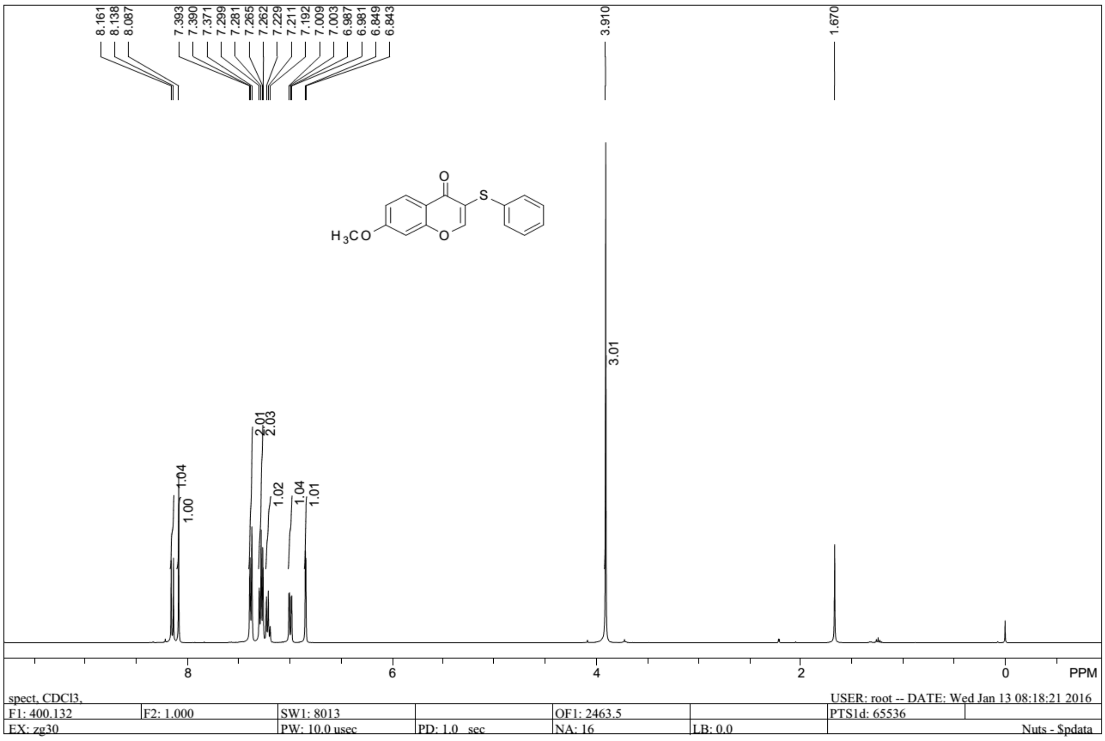

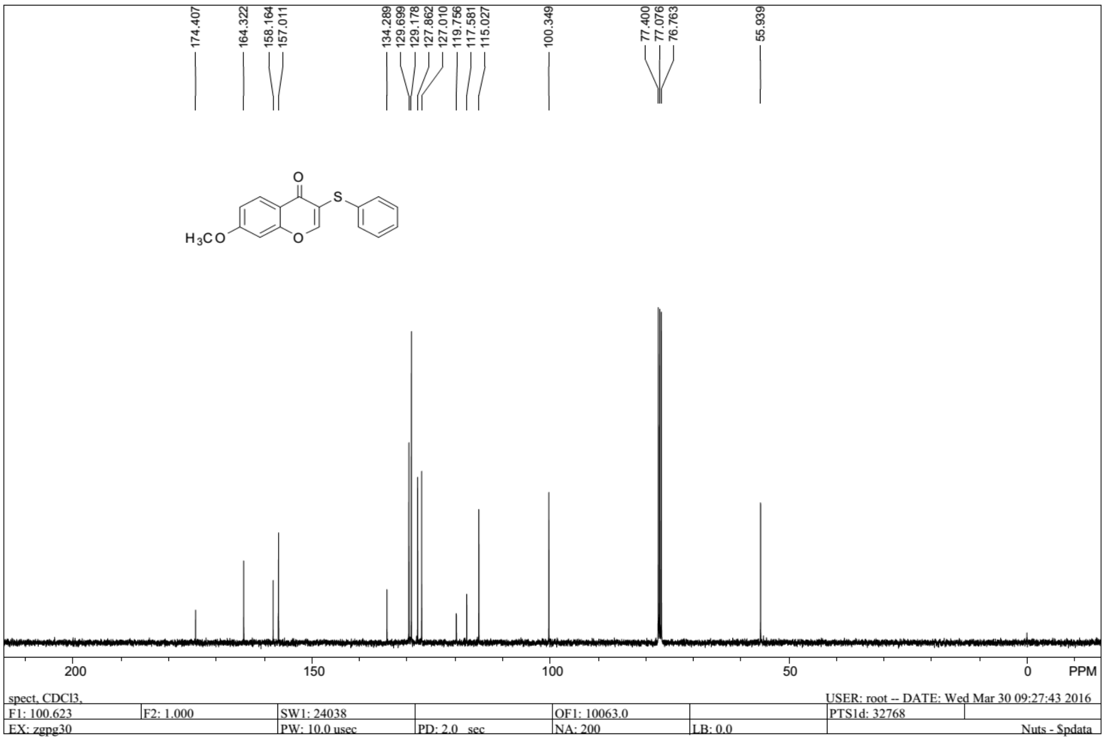

^{1}H and ^{13}C NMR spectra of **3b**

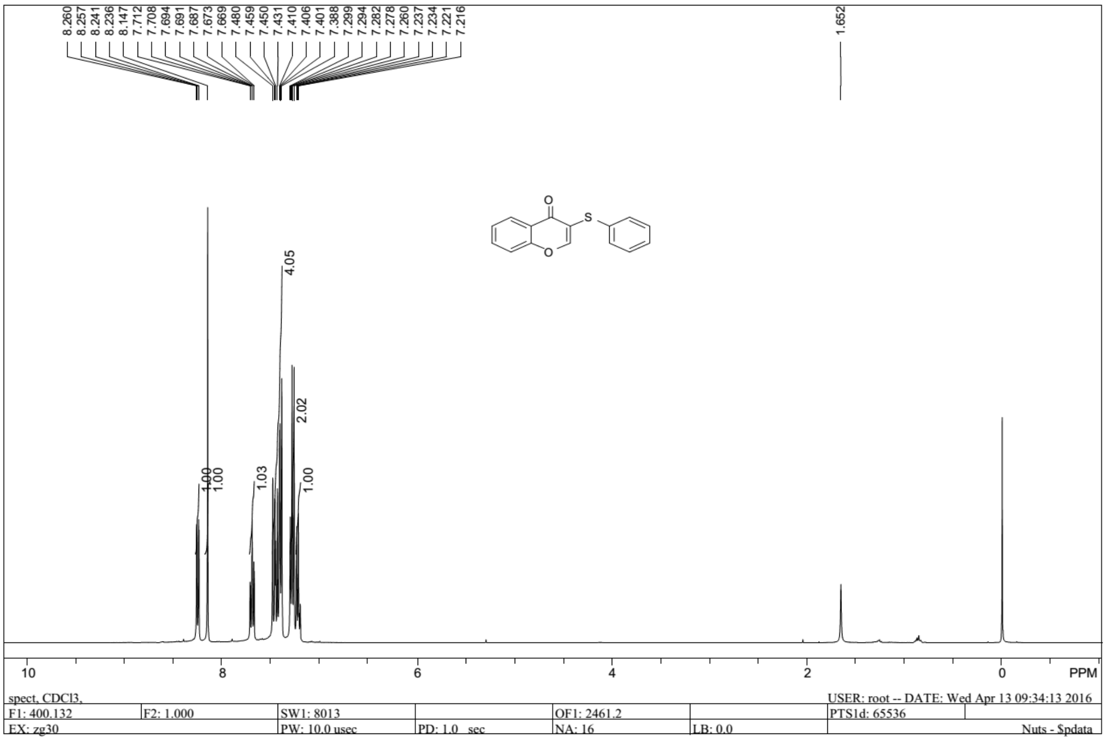

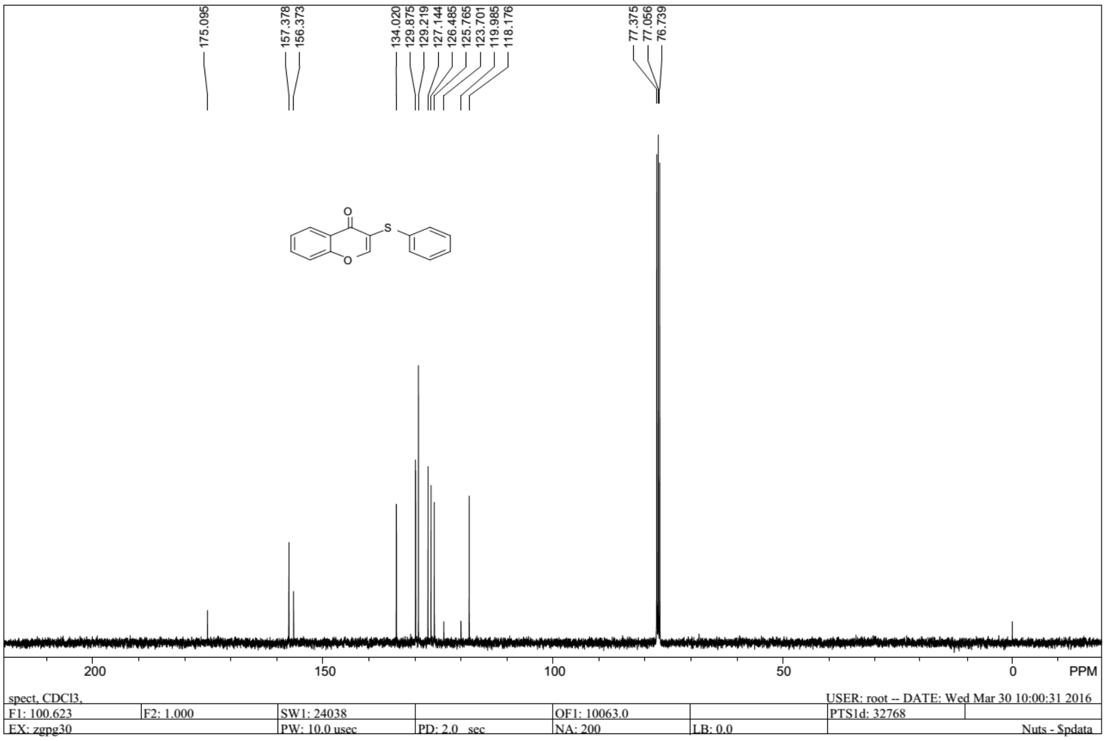

^{1}H and ^{13}C NMR spectra of **3c**

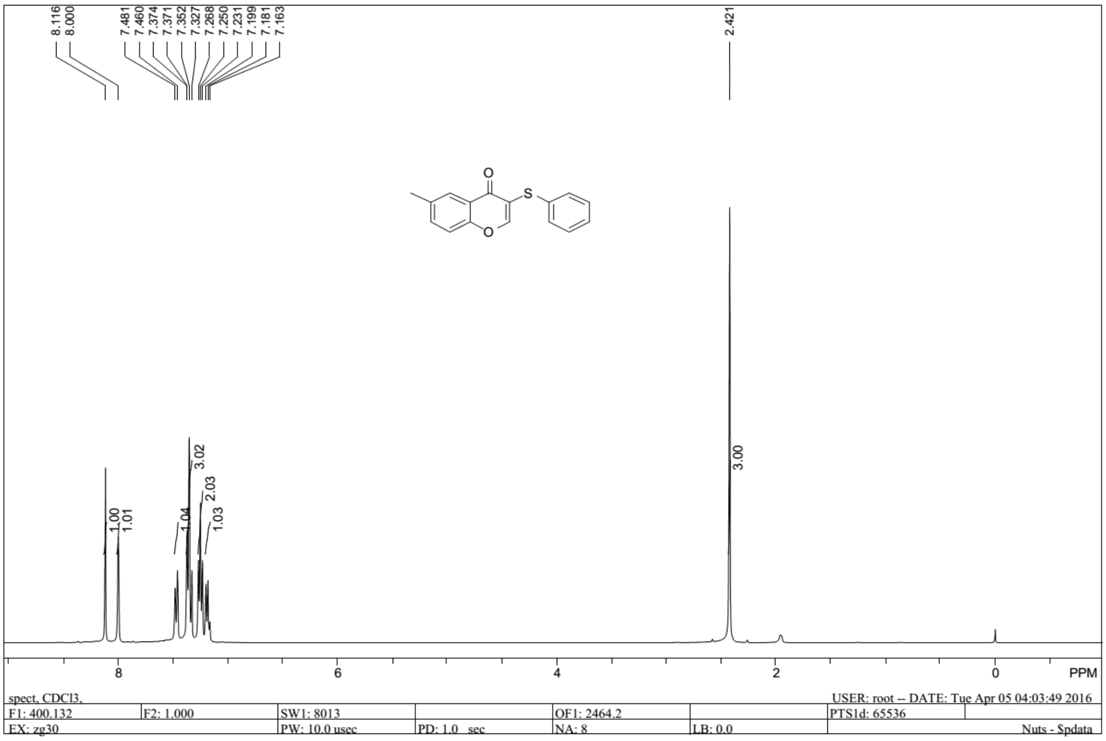

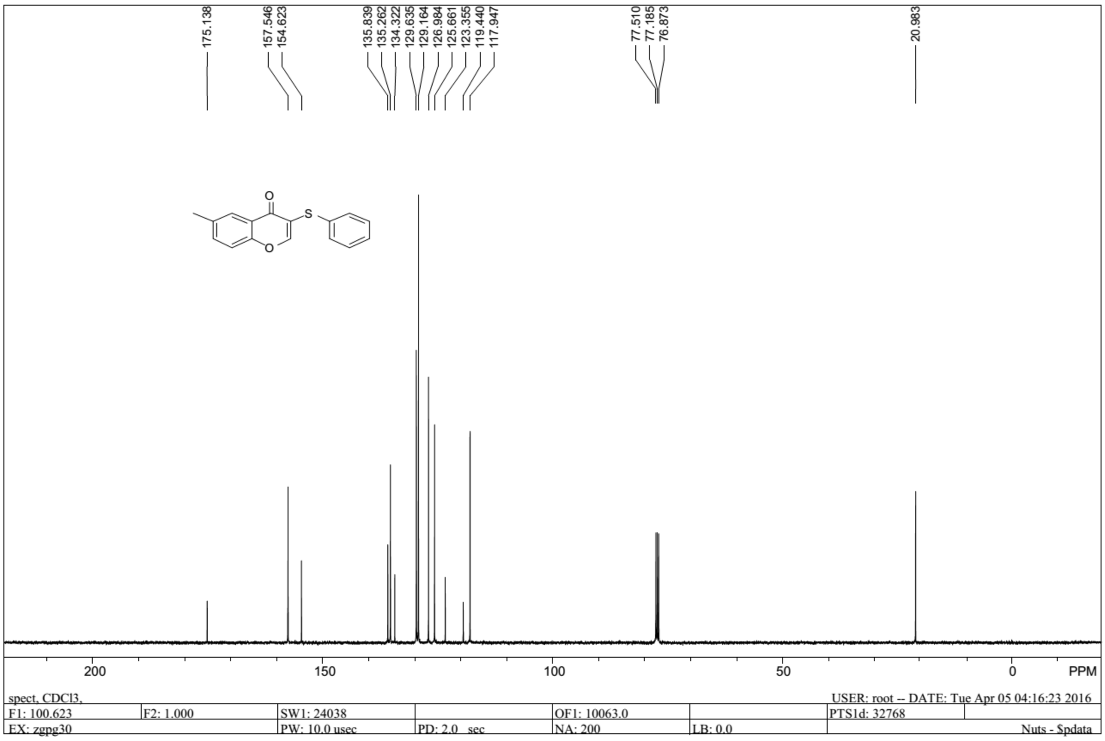

^{1}H and ^{13}C NMR spectra of **3d**

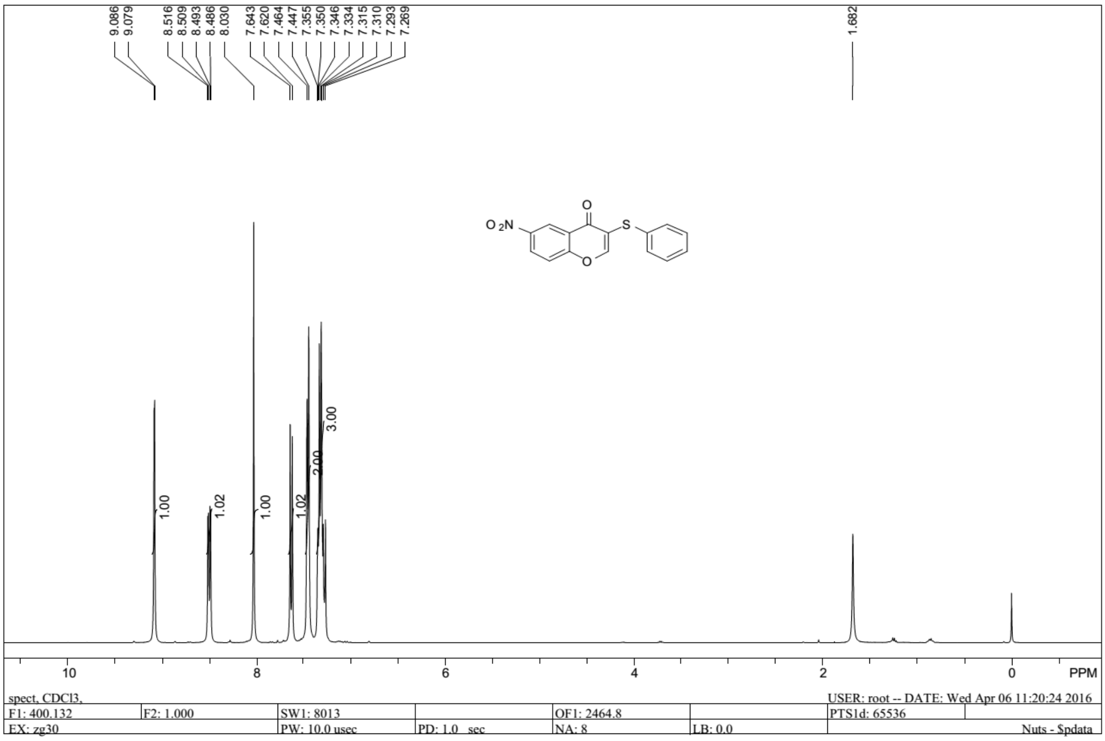

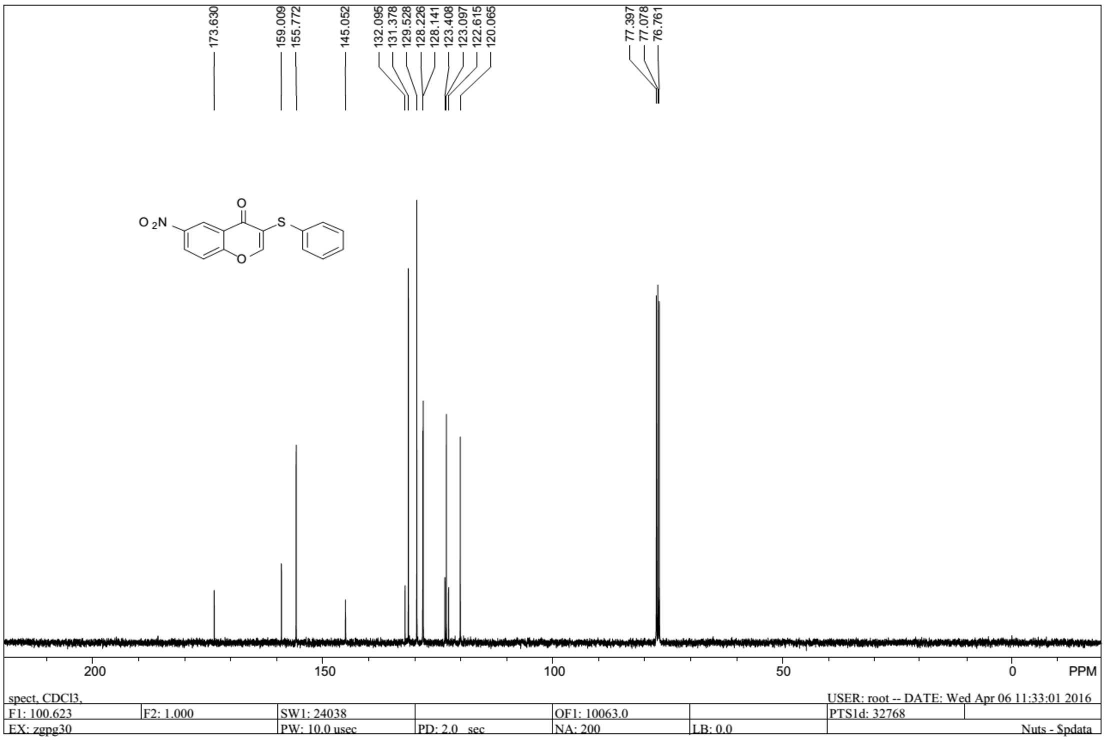

^{1}H and ^{13}C NMR spectra of **3e**

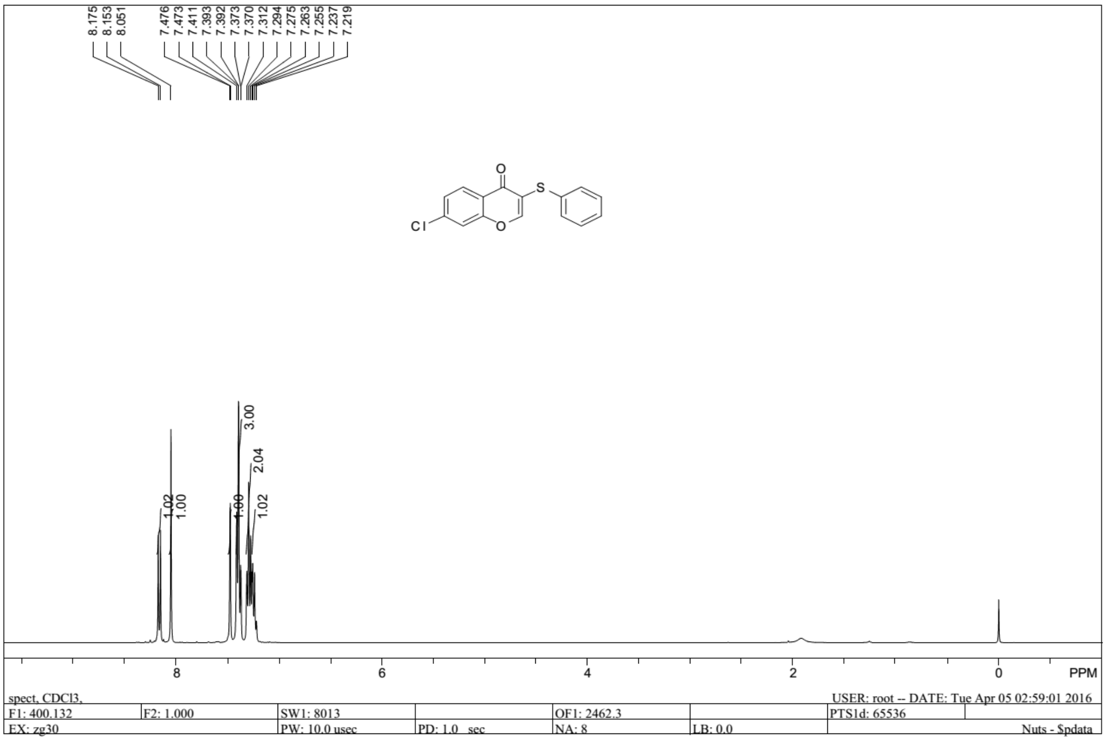

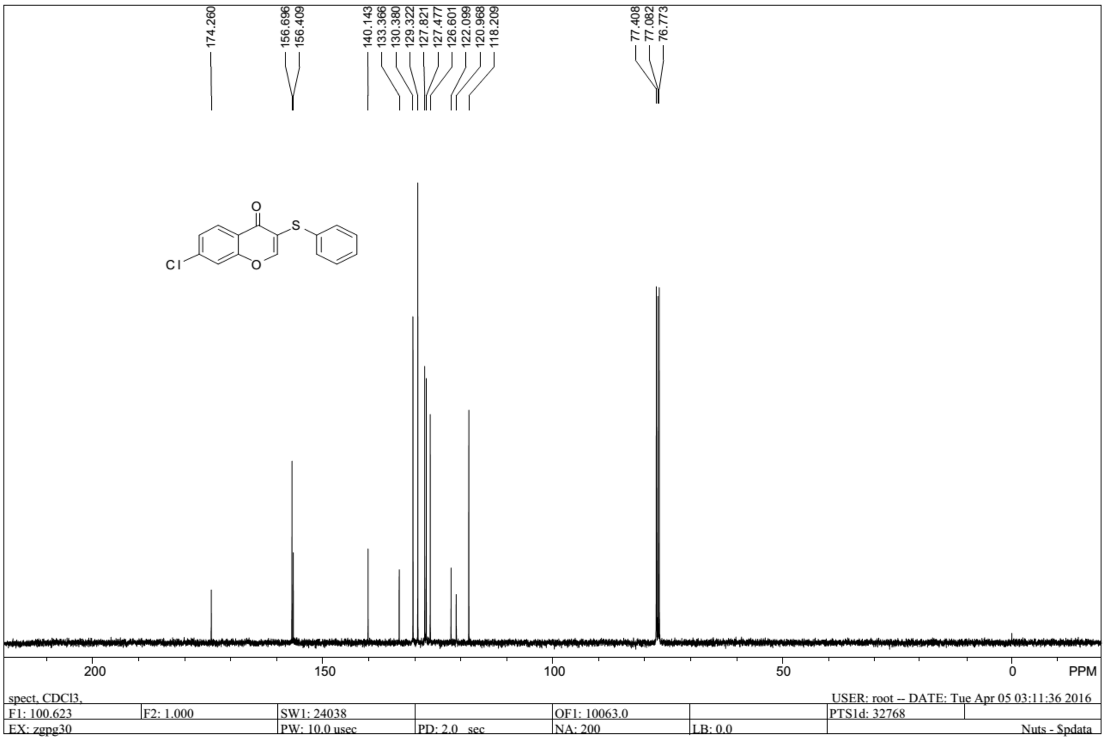

^{1}H and ^{13}C NMR spectra of **3f**

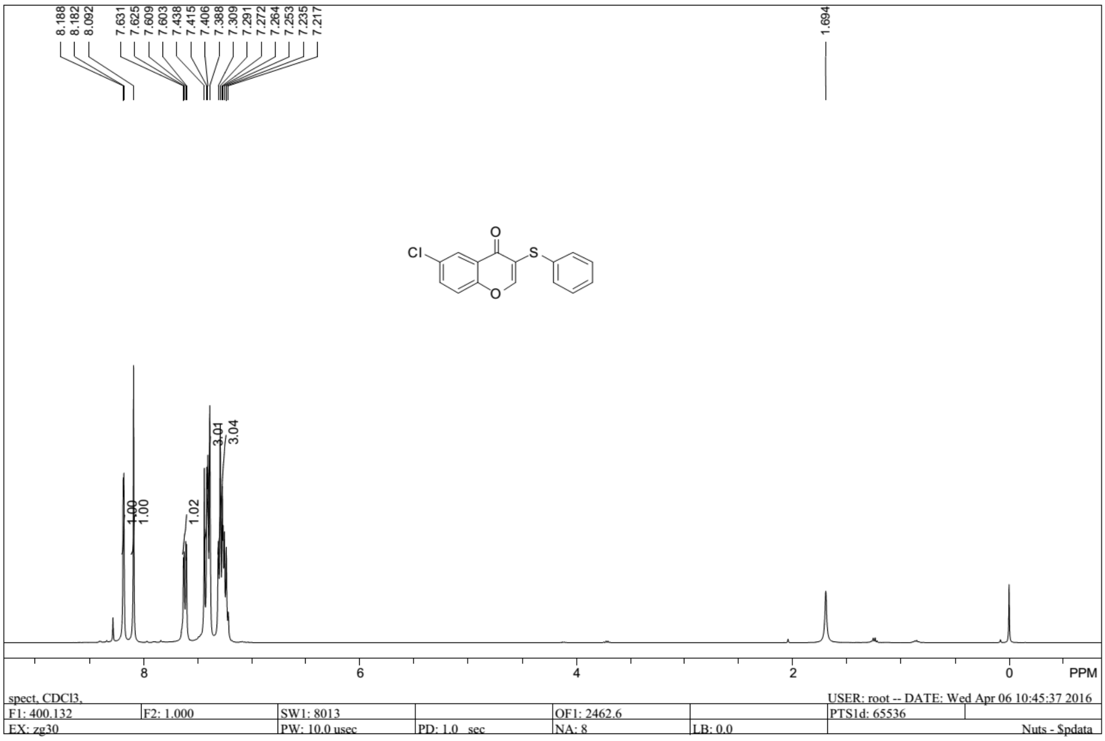

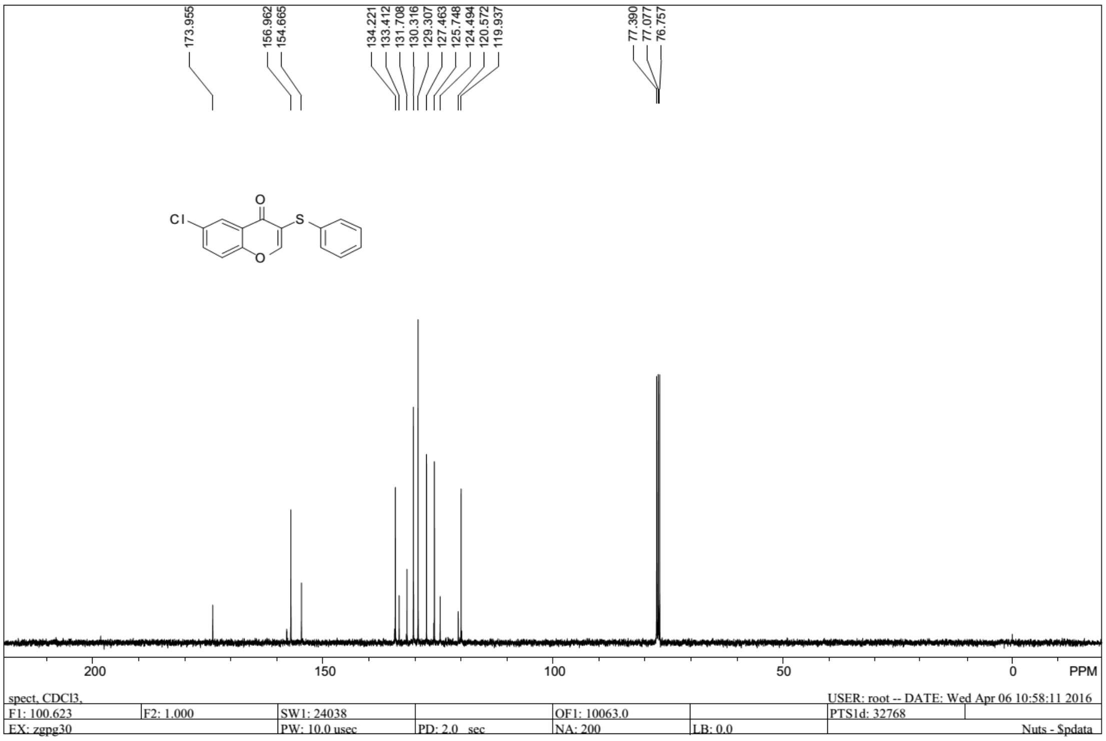

^{1}H and ^{13}C NMR spectra of **3g**

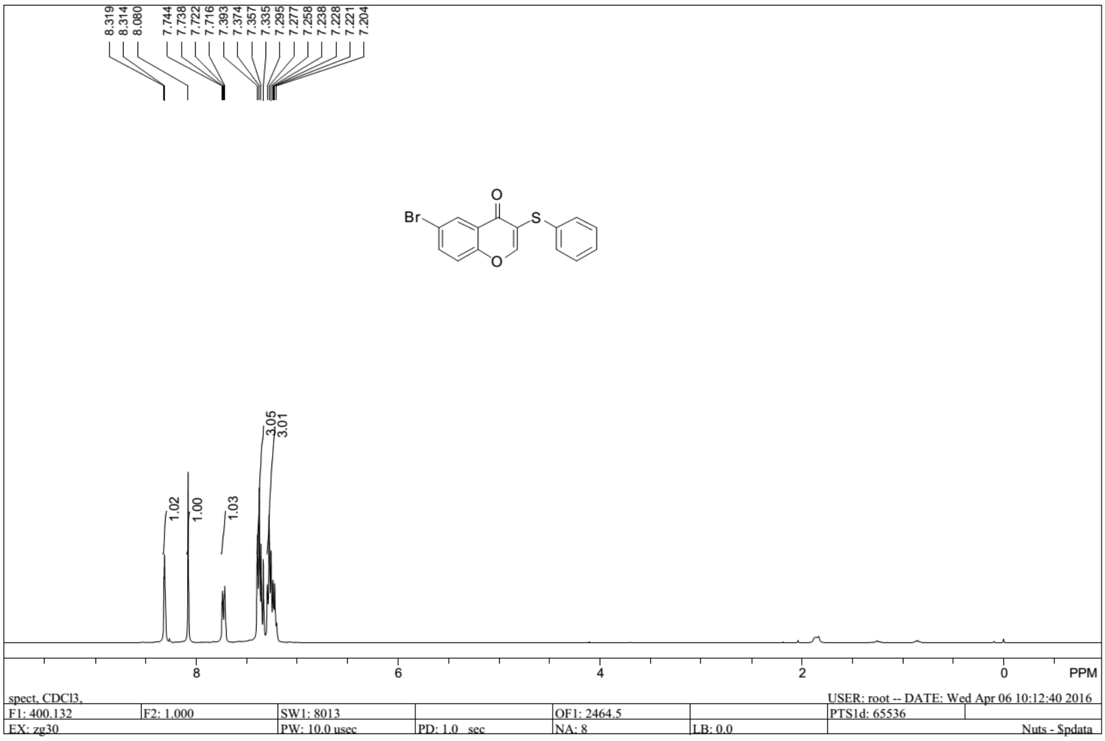

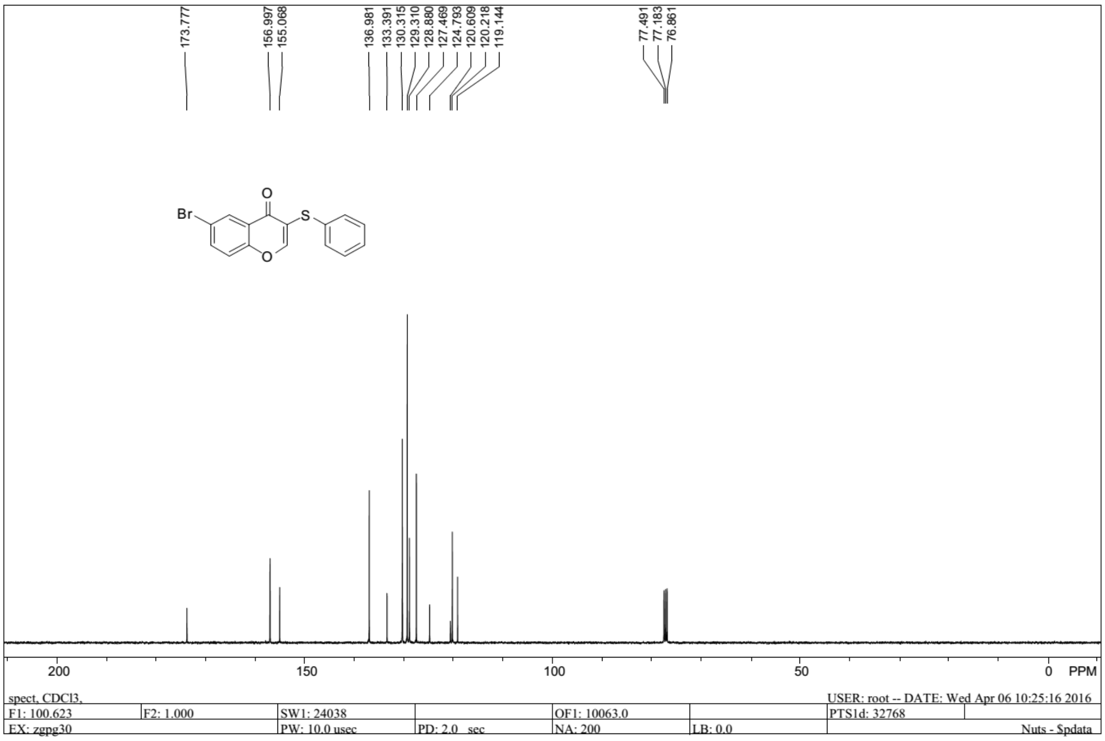

^1H and ^{13}C NMR spectra of **3h**

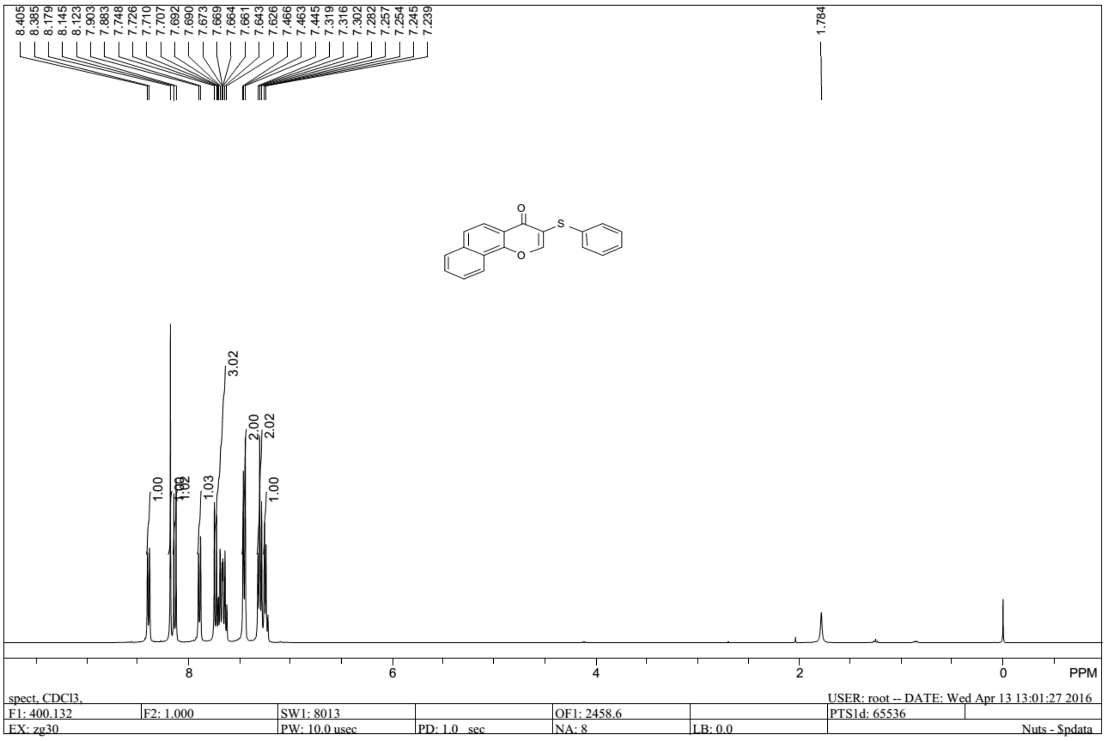

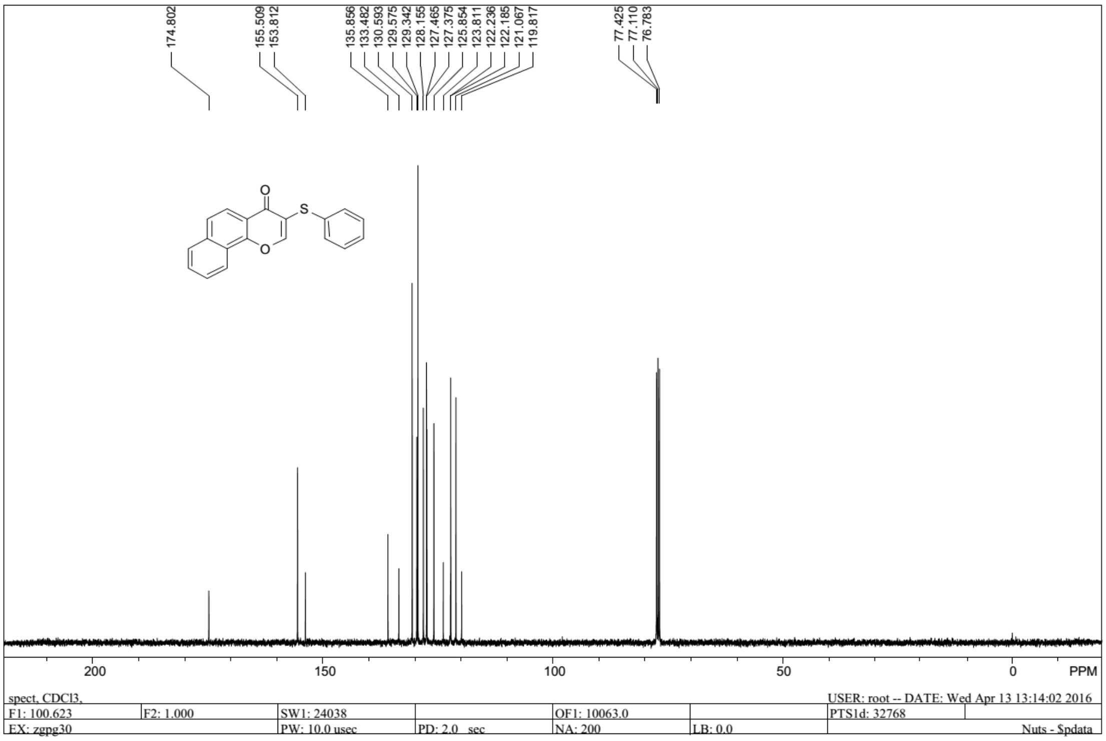

^{1}H and ^{13}C NMR spectra of **3i**

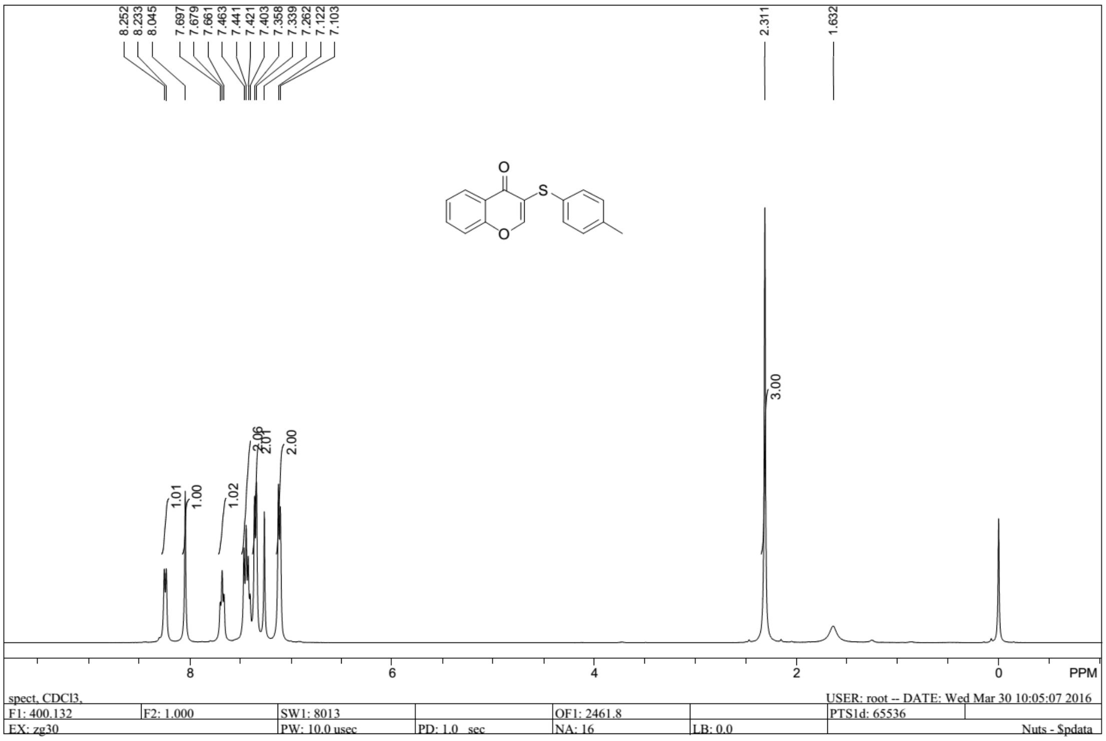

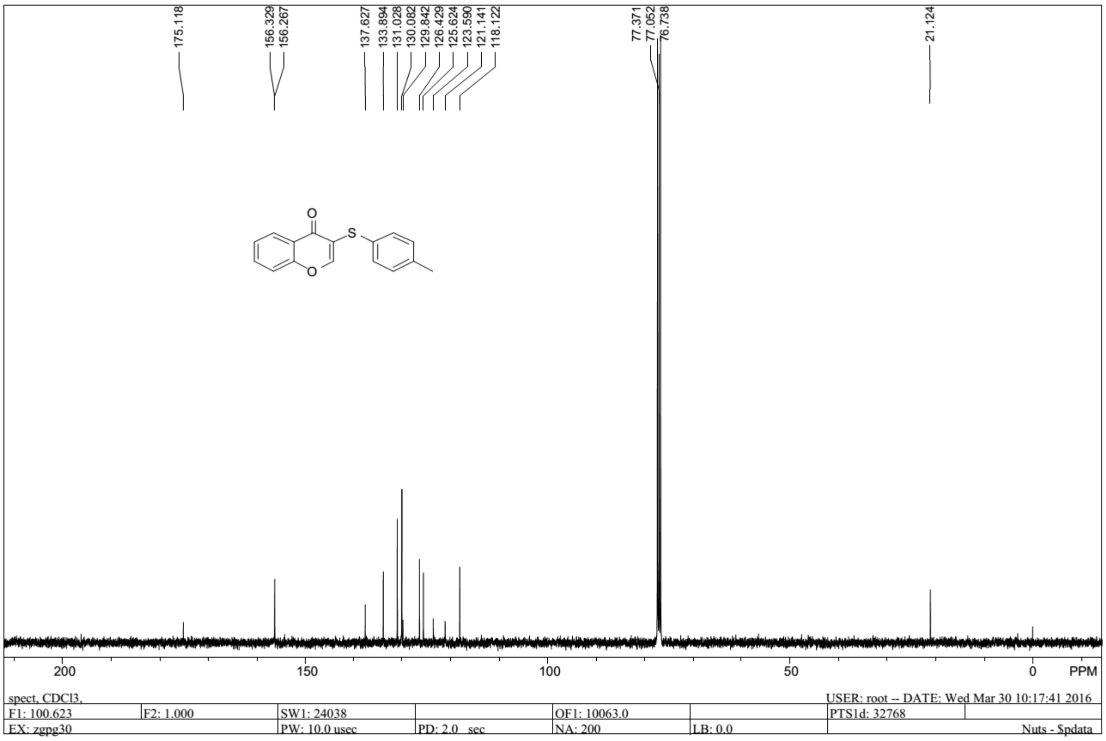

^{1}H and ^{13}C NMR spectra of **3j**

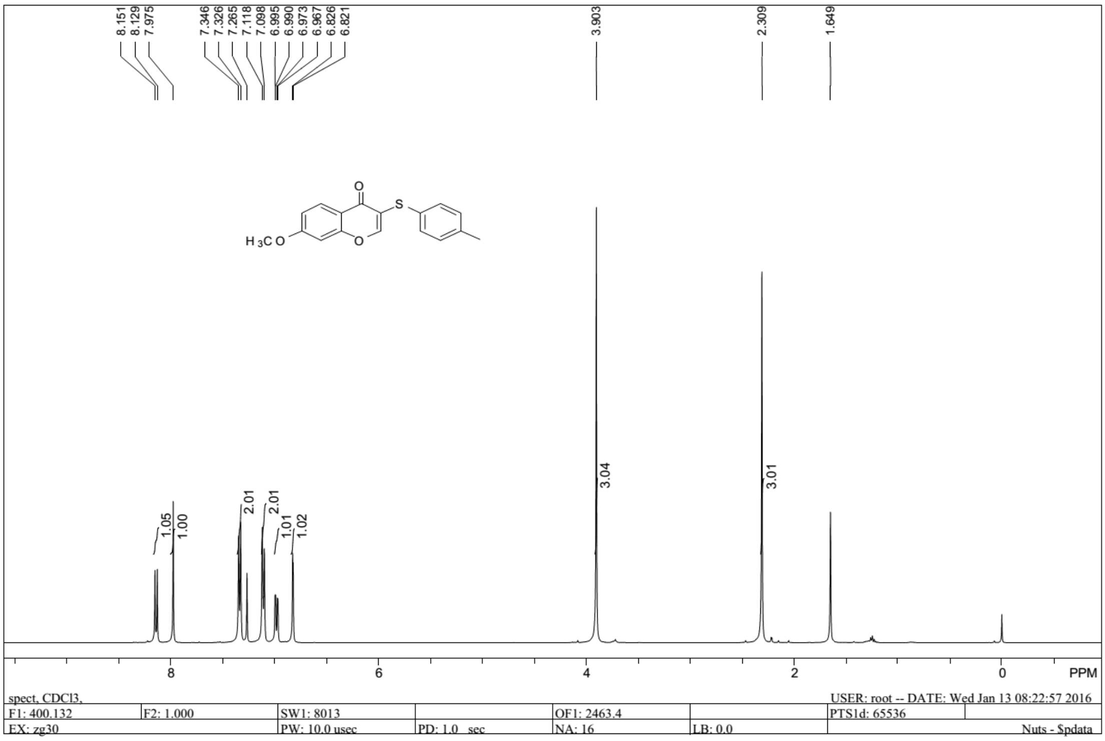

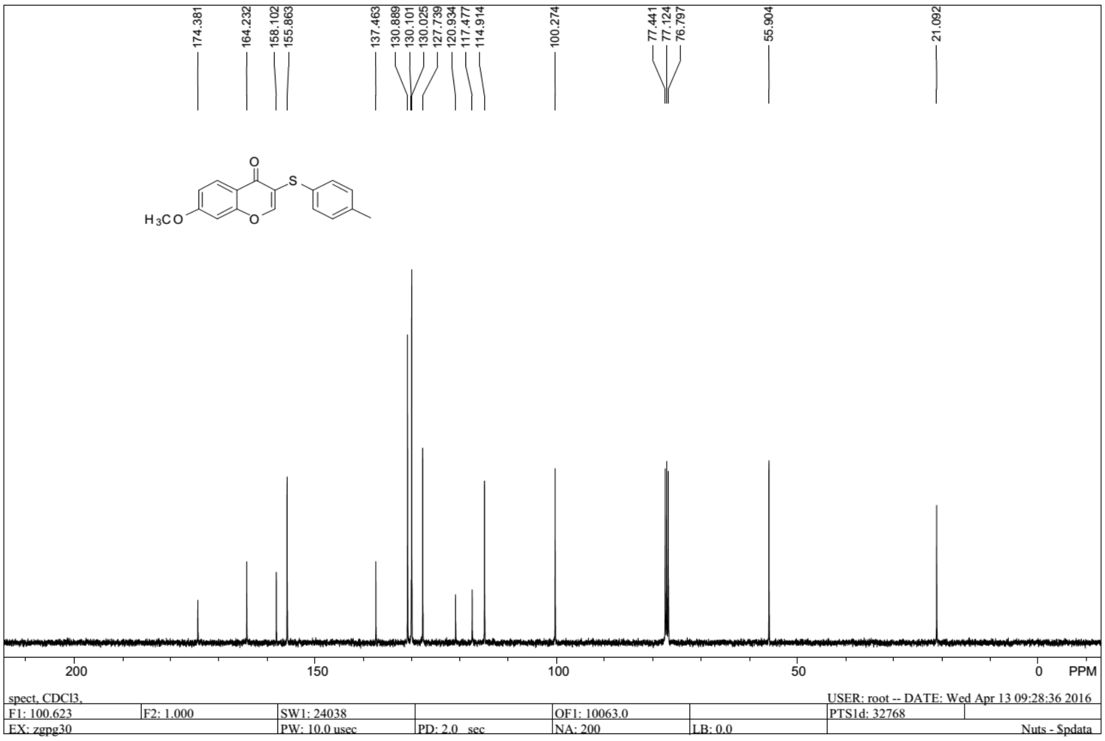

^{1}H and ^{13}C NMR spectra of **3k**

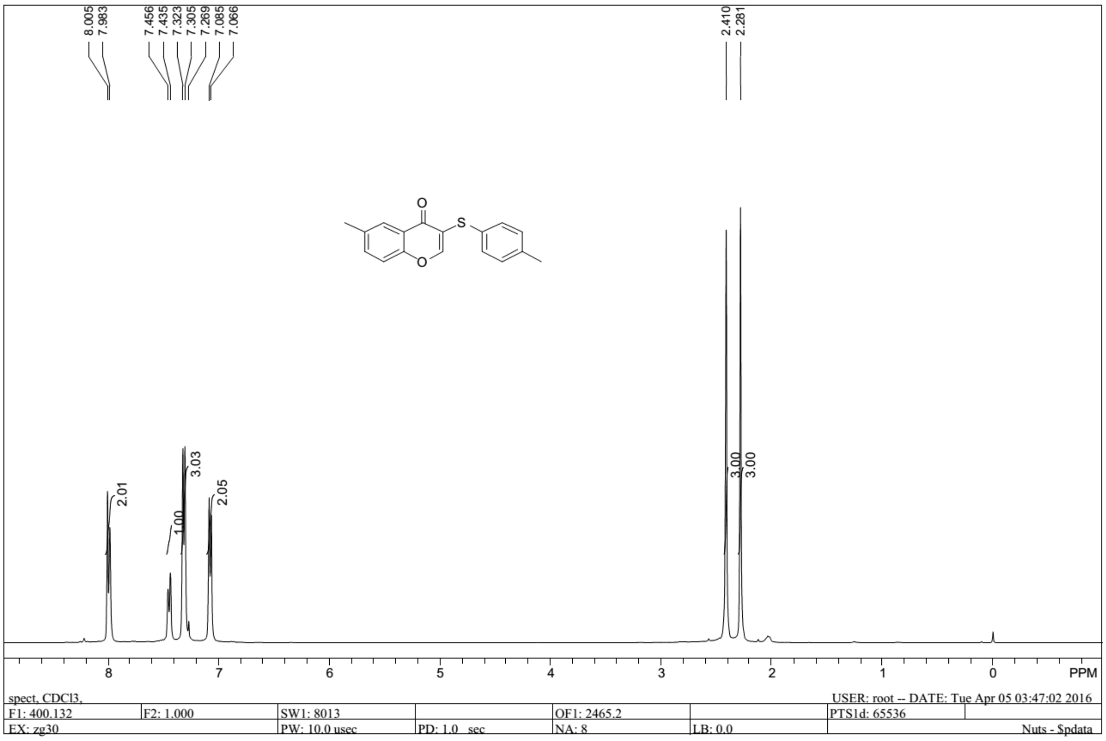

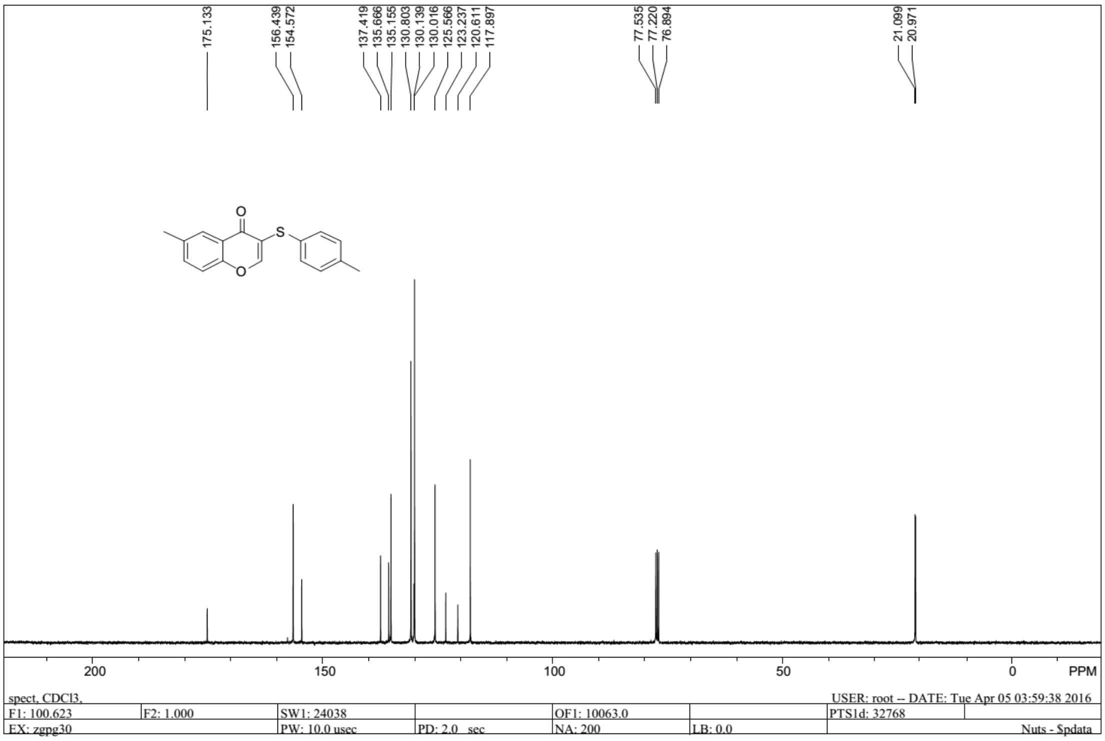

^{1}H and ^{13}C NMR spectra of **3l**

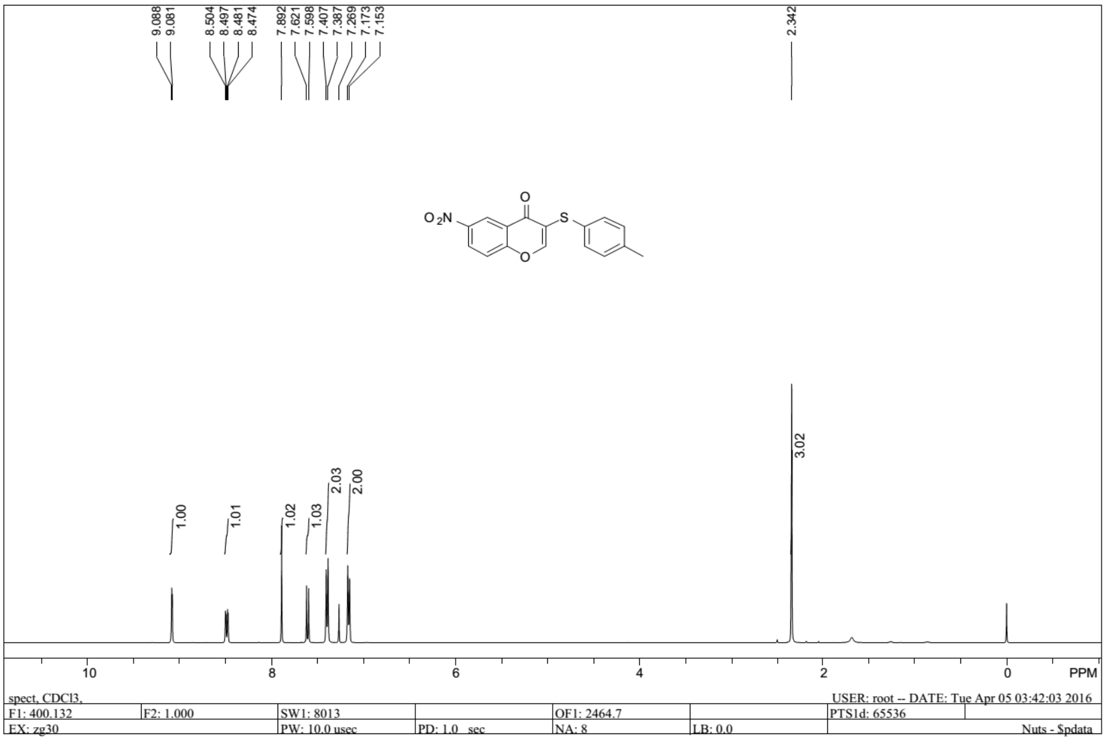

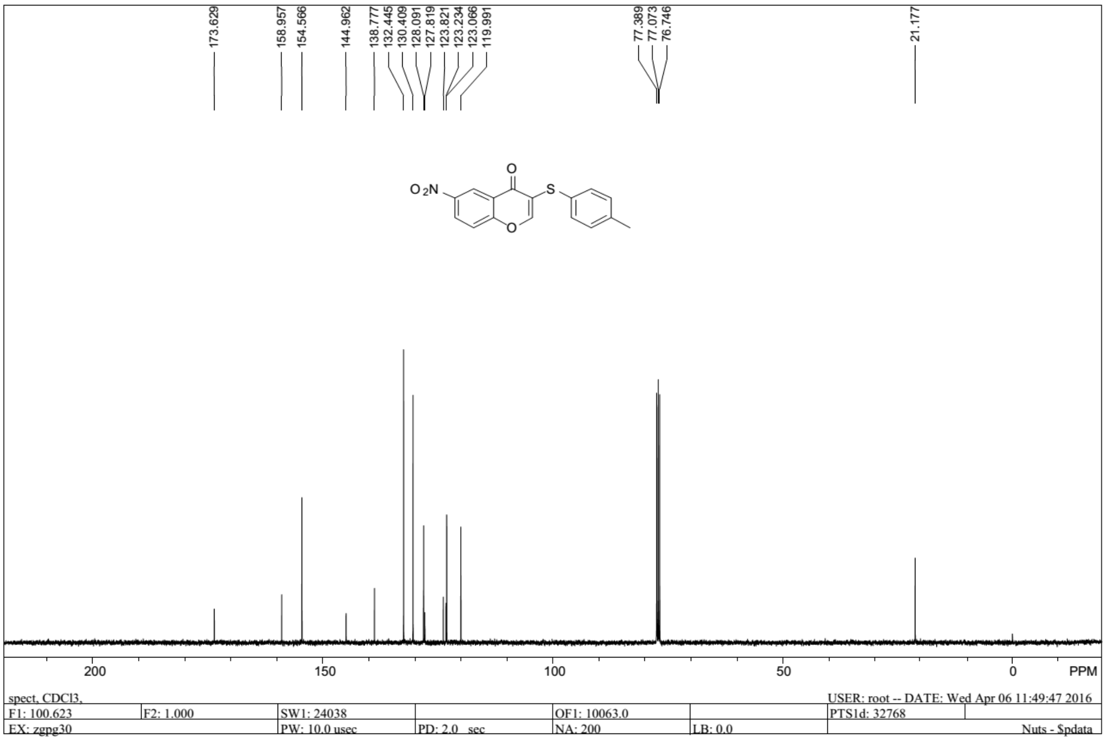

^{1}H and ^{13}C NMR spectra of **3m**

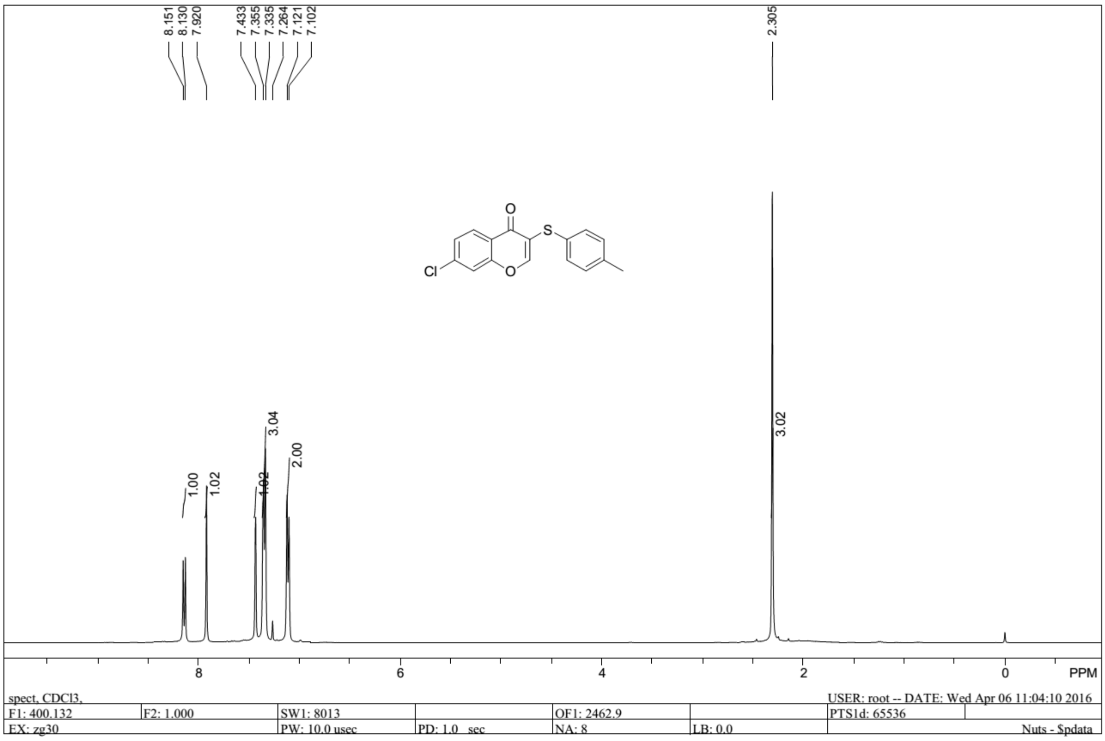

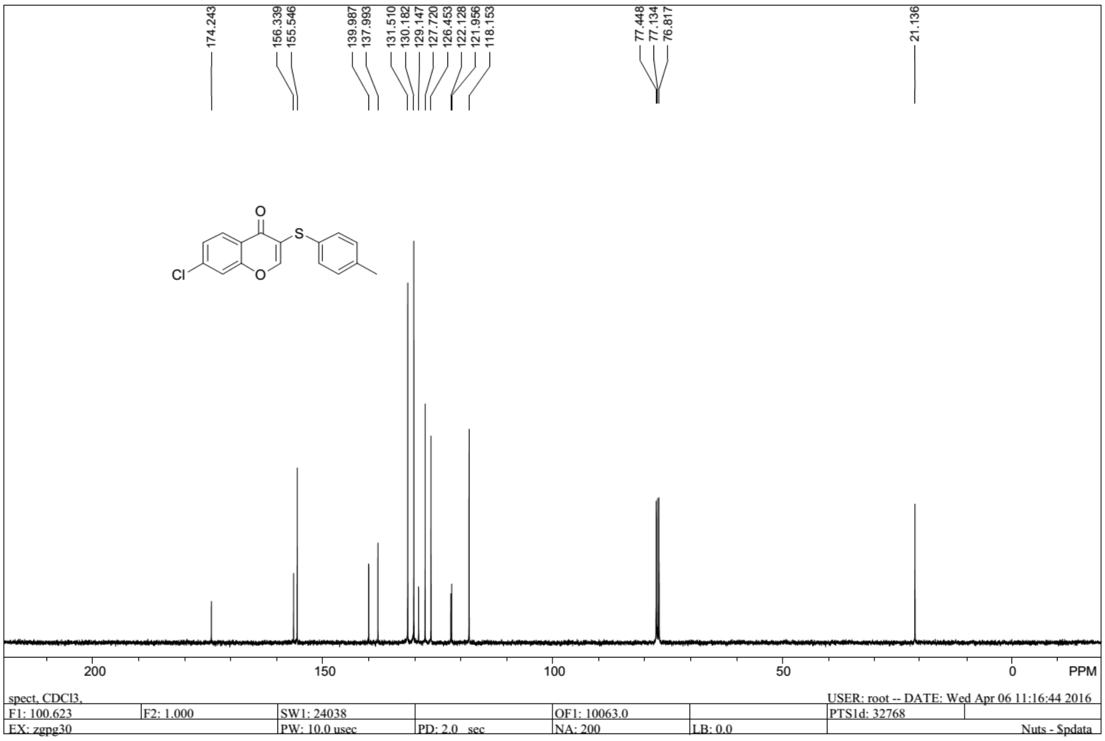

^{1}H and ^{13}C NMR spectra of **3n**

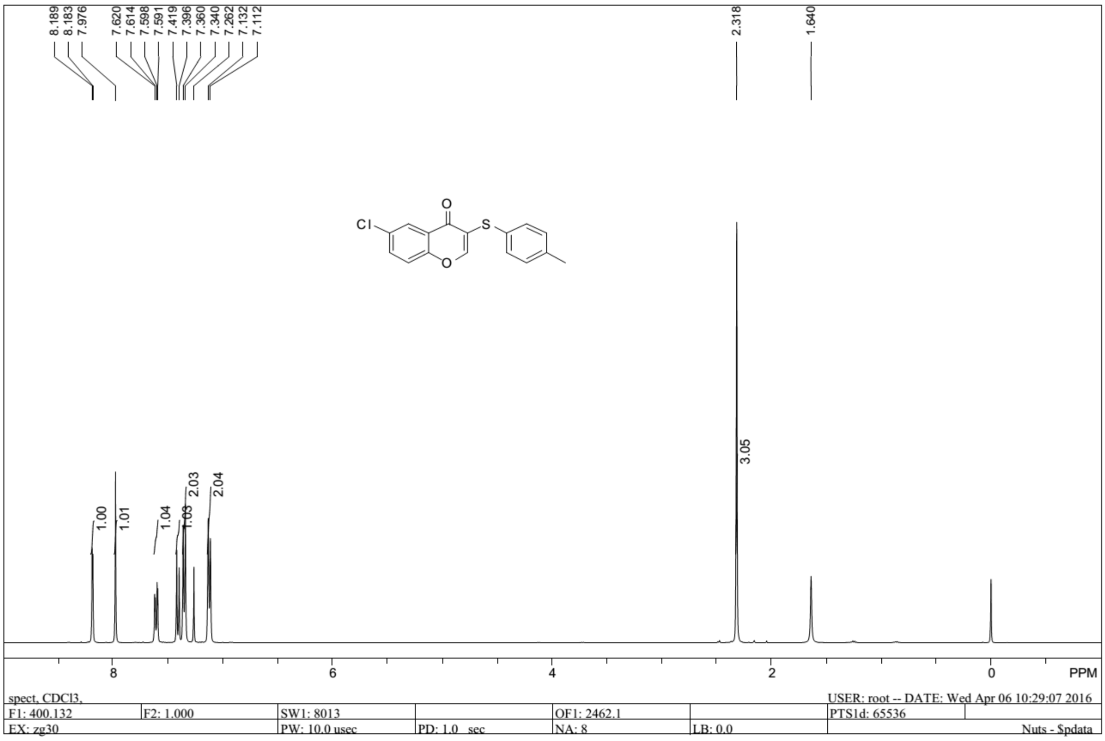

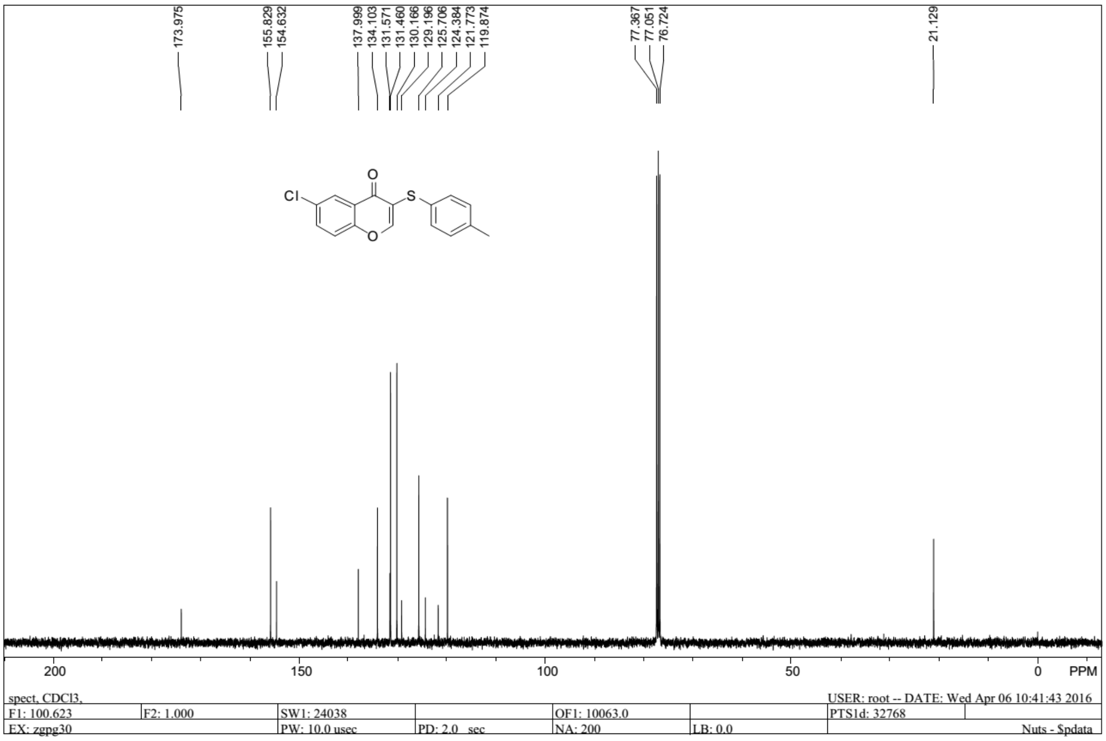

^{1}H and ^{13}C NMR spectra of **3o**

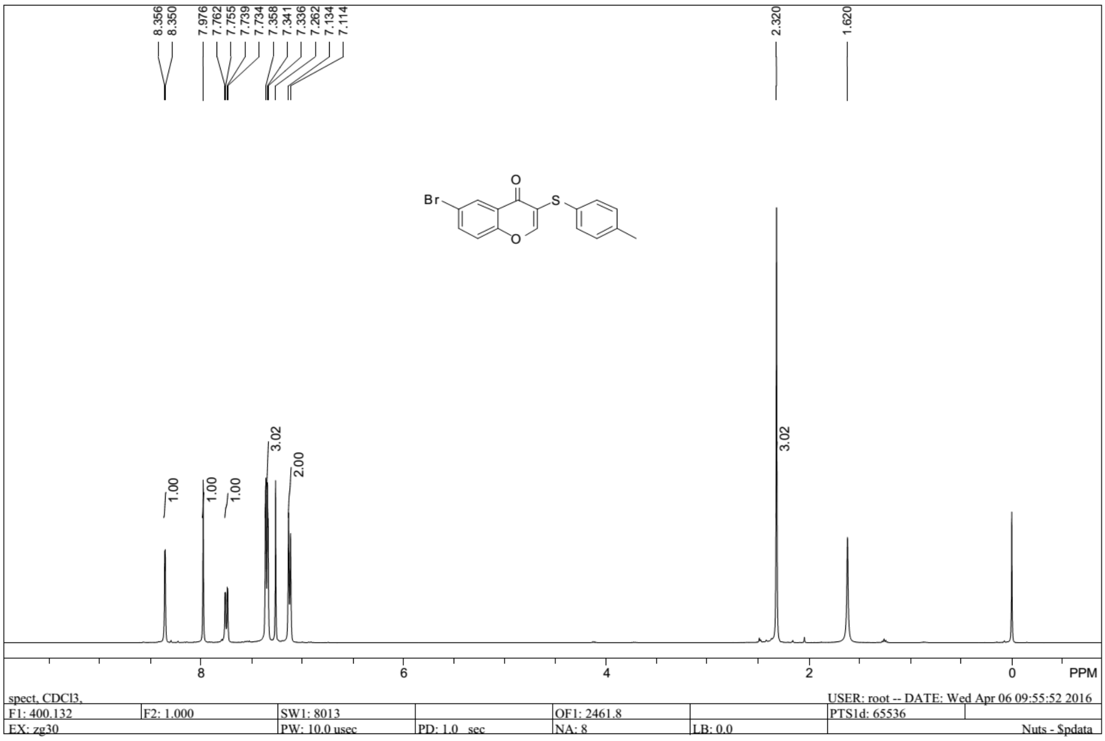

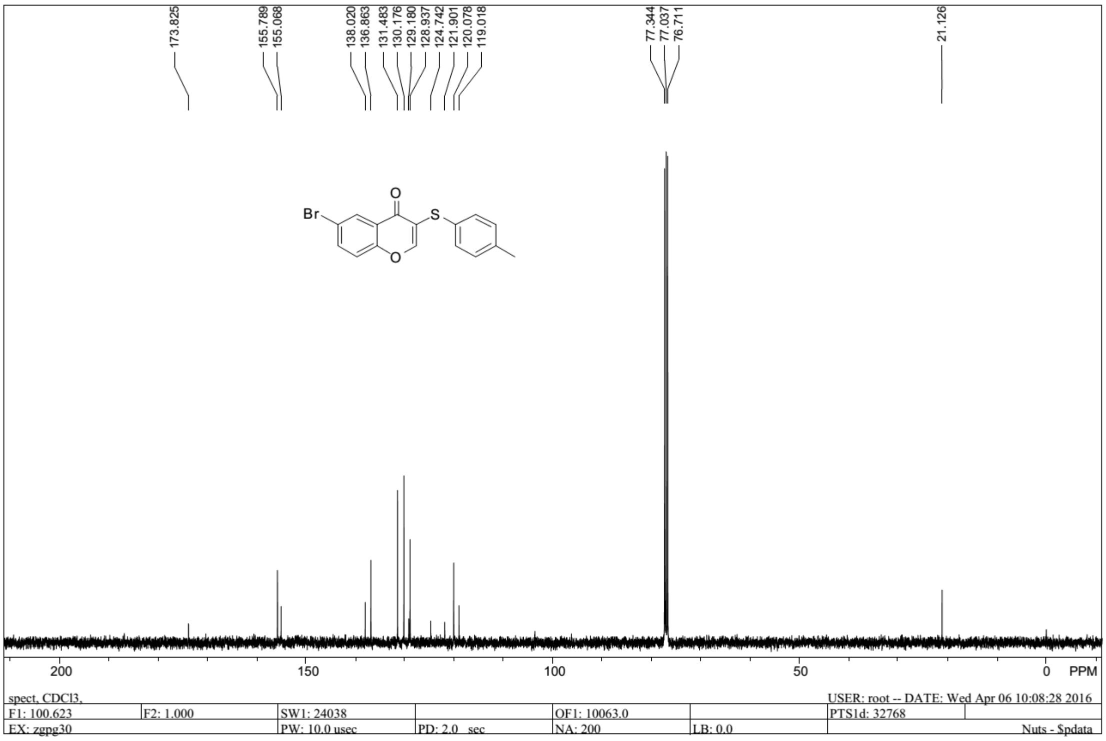

^{1}H and ^{13}C NMR spectra of **3p**

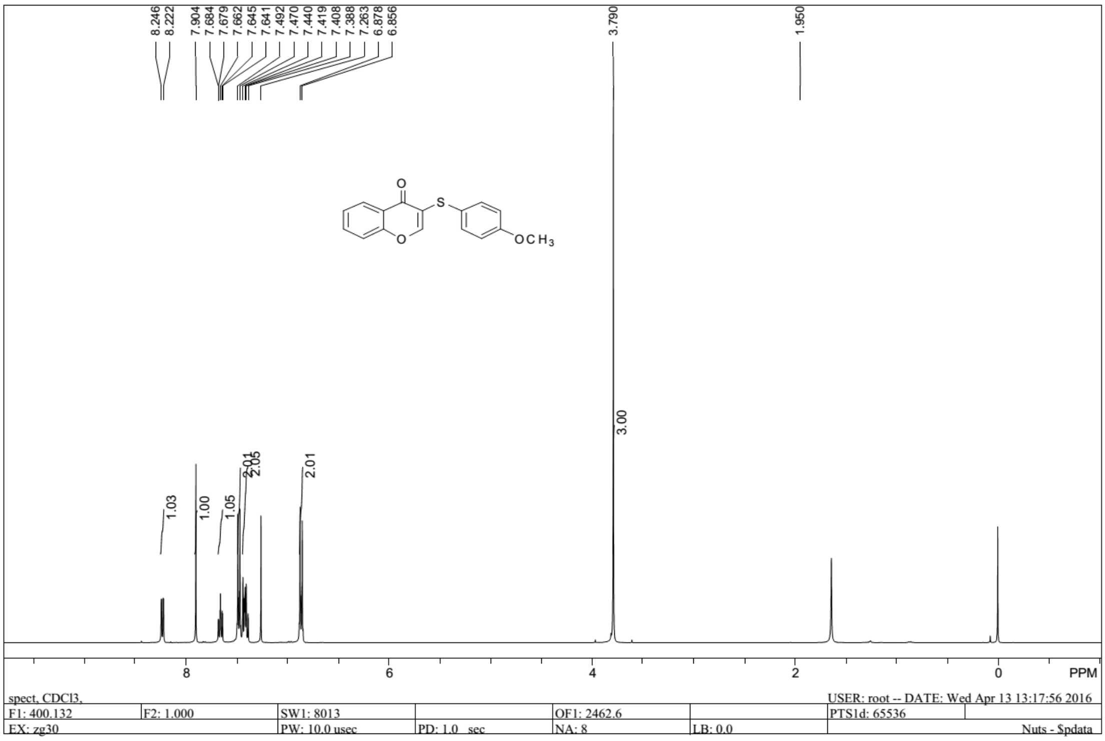

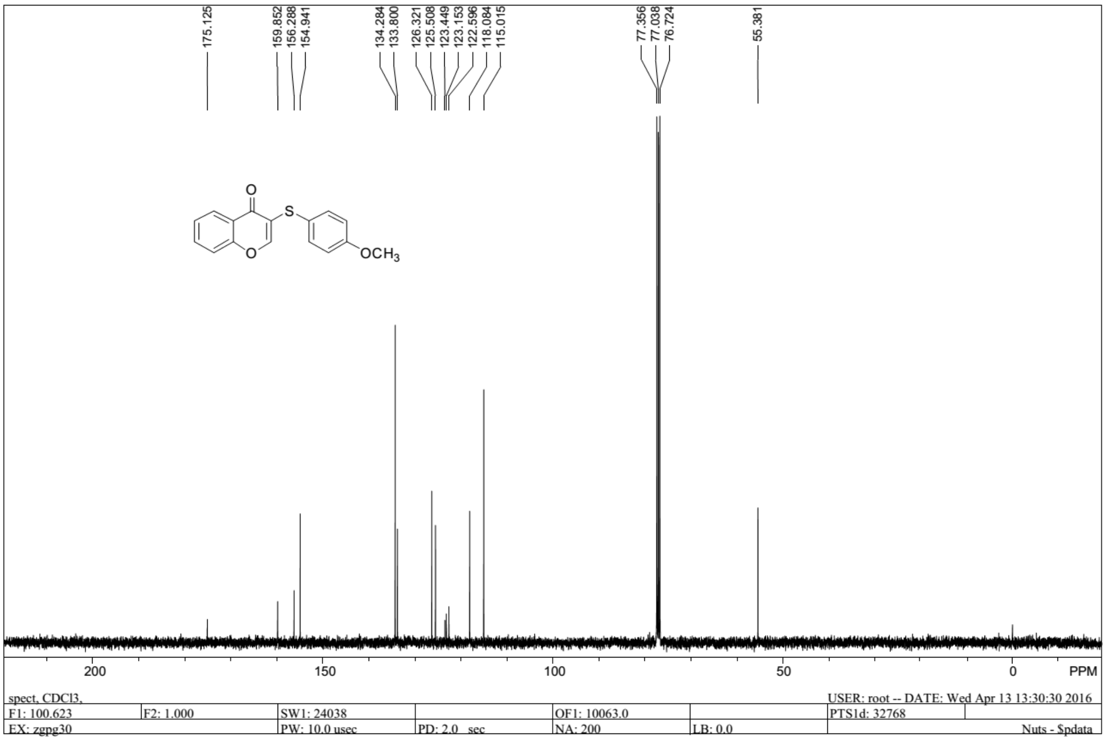

^{1}H and ^{13}C NMR spectra of **3q**

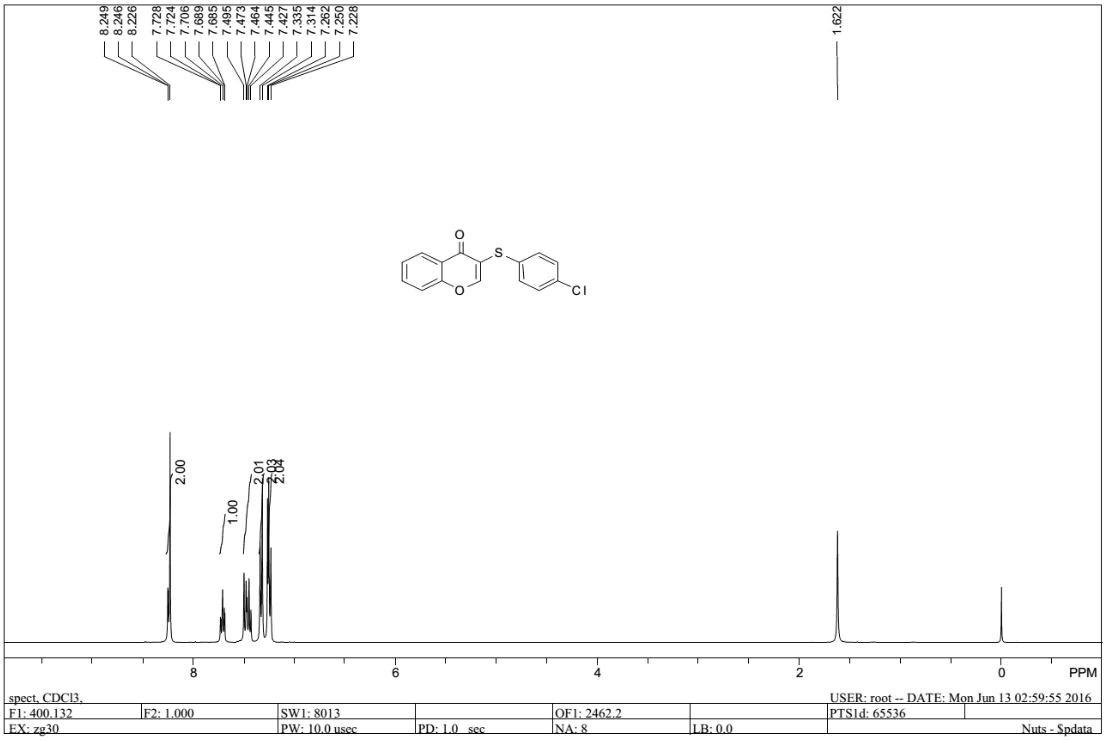

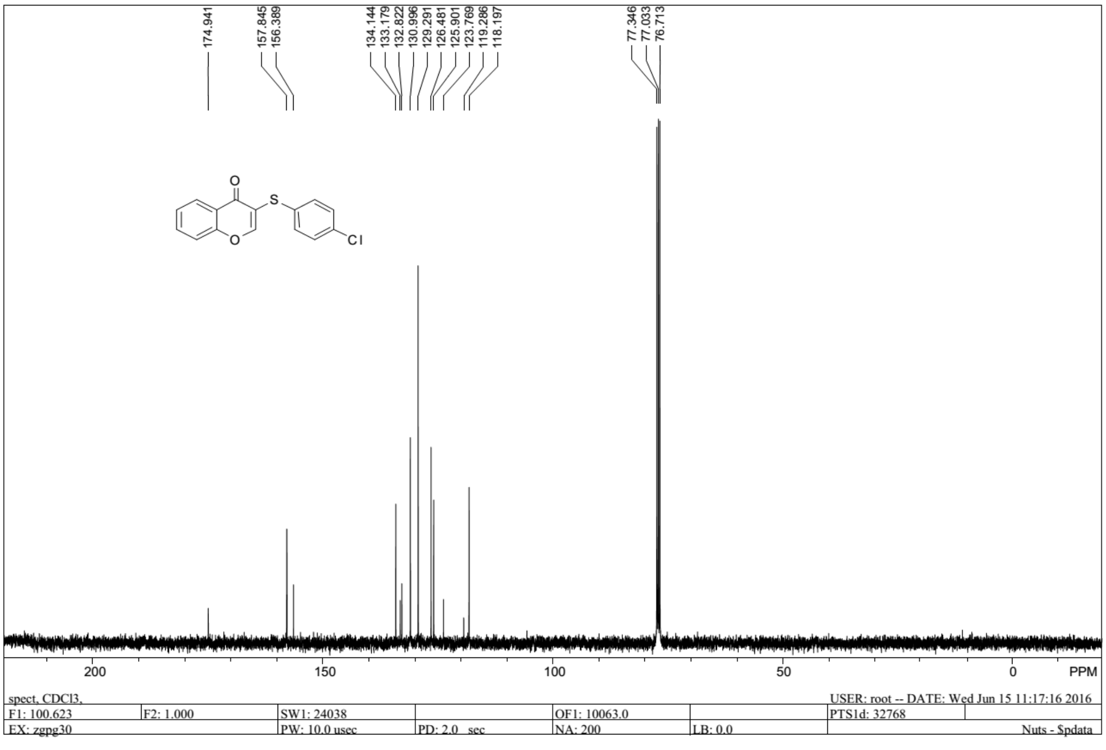

^{1}H and ^{13}C NMR spectra of **3r**

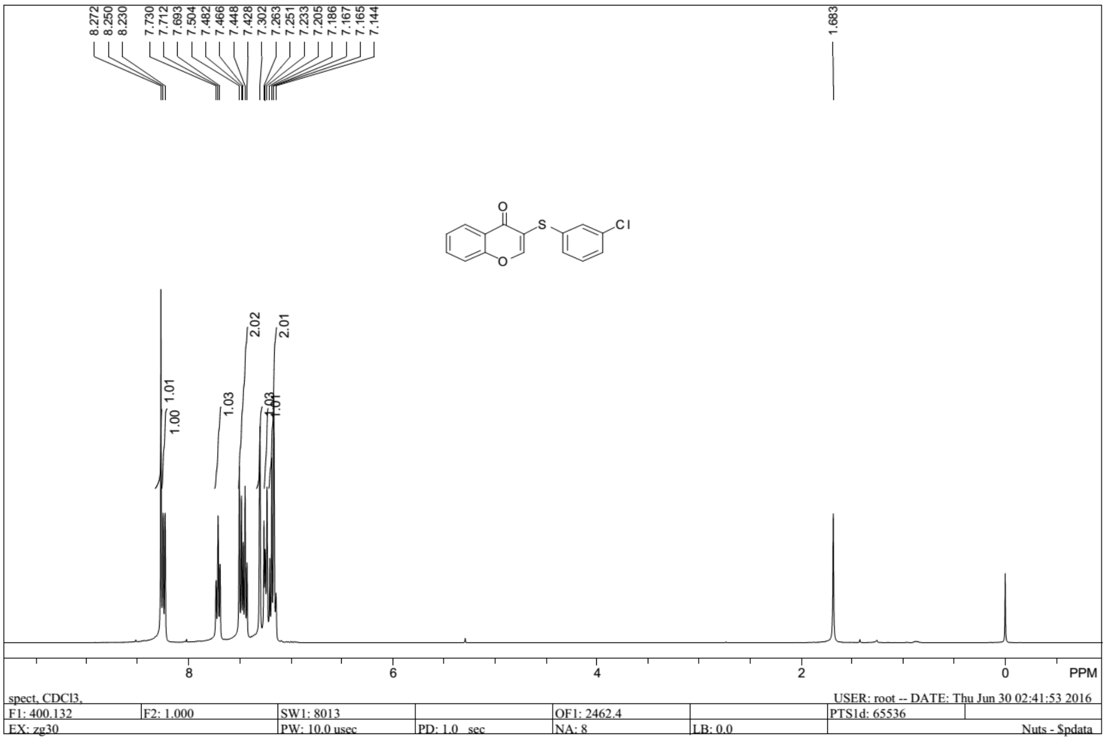

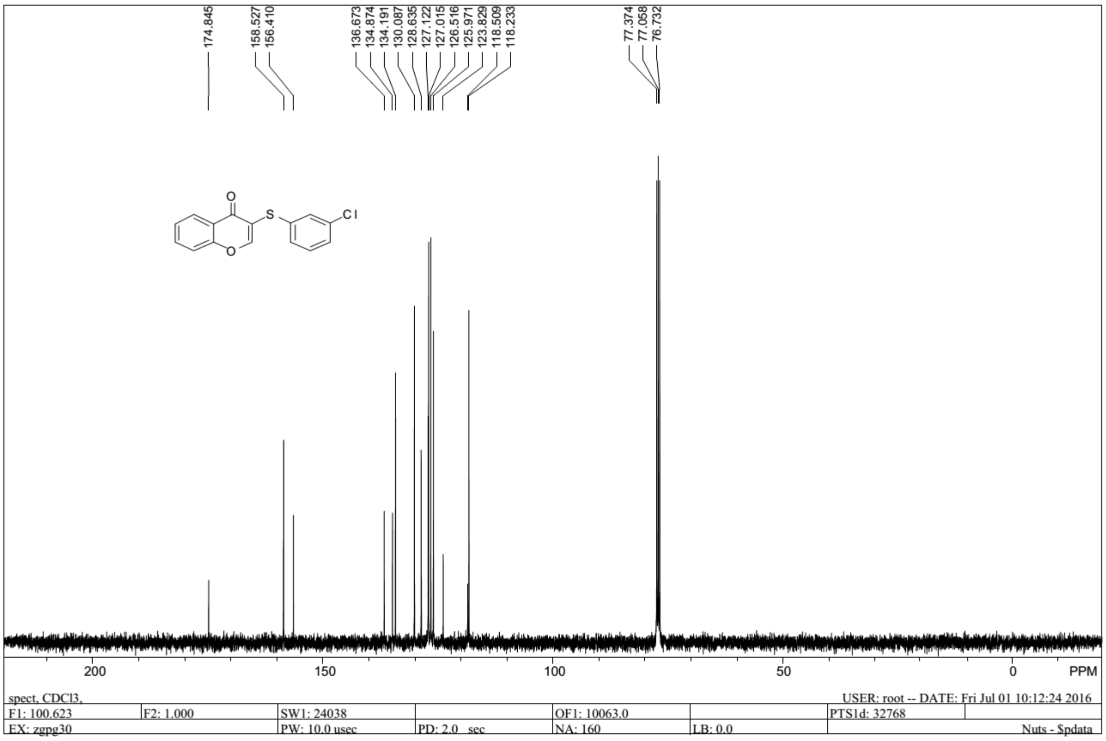

^{1}H and ^{13}C NMR spectra of **3s**

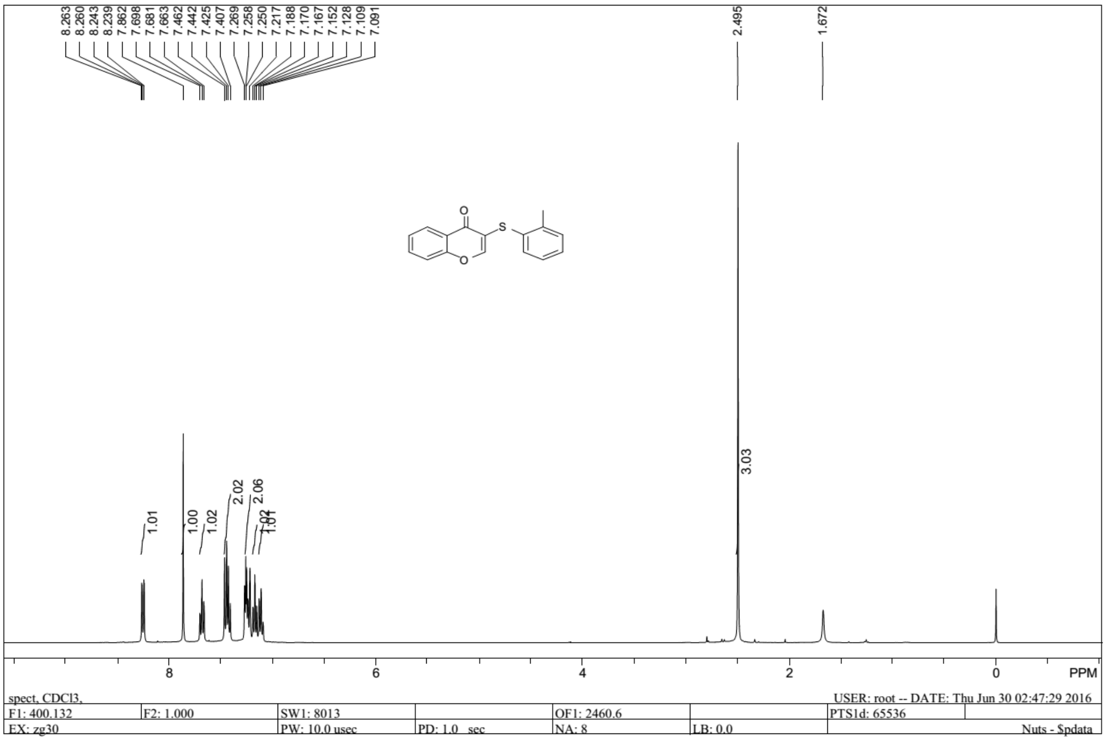

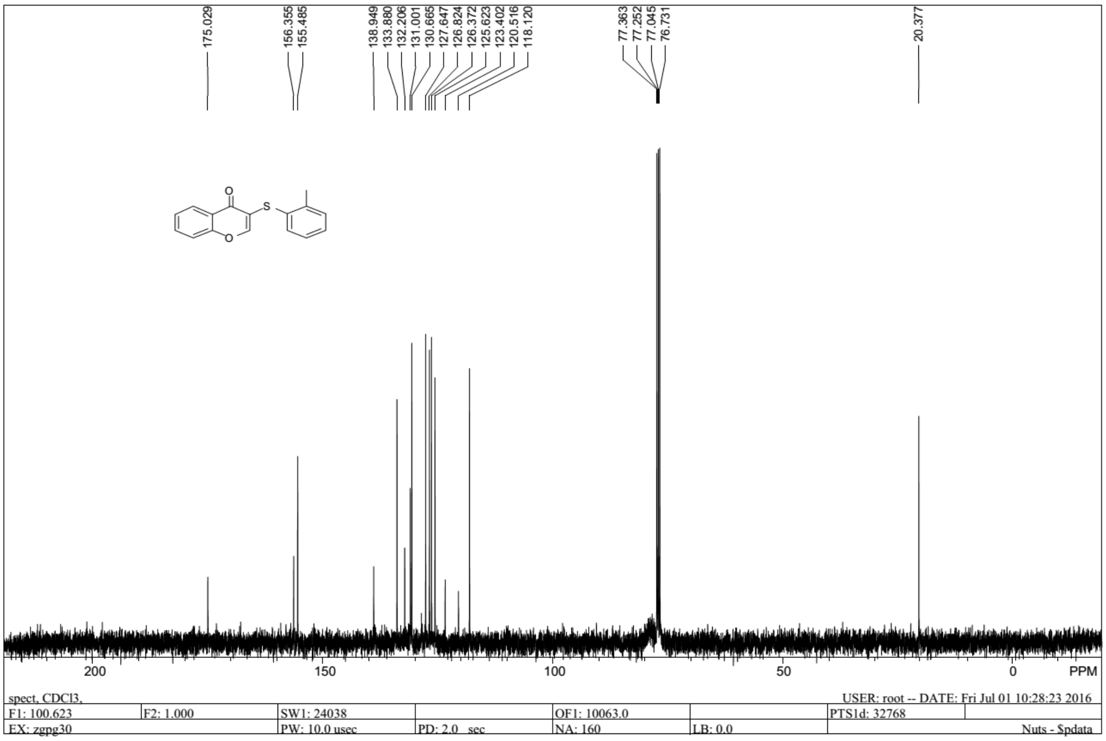

Supplement: File 1 — General experimental information, experimental details on the synthesis of products 3; full characterization data as well as 1H/13C NMR spectra of all products. [file Beilstein_J_Org_Chem-13-2017-s001.pdf]
